# Road Subgrade Stabilization Using Ground Granulated Blast Furnace Slag: Pavement Design, Cost Optimization, and CO_2_ Emission Reduction

**DOI:** 10.3390/ma19214639

**Published:** 2026-10-31

**Authors:** Bahadır Karabaş, Ali Ulvi Uzer

**Affiliations:** 1 Department of Civil Engineering, İnönü University, Malatya 44280, Türkiye; 2 Vocational School of Technical Sciences, Kayseri University, Kayseri 38280, Türkiye; aliulviuzer@kayseri.edu.tr

**Keywords:** GGBFS, subgrade stabilization, CO_2_ emissions, sustainability, transportation geotechnics

## Abstract

This study examines the effects of using GGBFS in road subgrade stabilization on pavement structure and sustainability. Samples were prepared by adding GGBFS at ratios of 5%, 10%, 15%, and 20% to BT (Bentonite) soil samples evaluated as road subgrade. As a result of the laboratory experiments, the optimum GGBFS ratio was determined as 15%; this ratio provided increases of 109%, 185%, and 234% in California Bearing Ratio (CBR) values at 5.0 mm penetration depth (adopted as the primary structural design benchmark) at the end of 3, 7, and 28 days of curing, respectively, while Unconfined Compressive Strength (UCS) values increased by 124%, 166%, and 208%. Pavement thickness and cost designs carried out according to the AASHTO 1993 method revealed a reduction of up to 29.41% in flexible pavement thickness and up to 14.20% in unit area cost. For rigid (concrete) pavement, a reduction of up to 6.06% in thickness and 6.77% in unit area cost was identified. For a road with dimensions of 1000 m × 20 m, cost savings of up to 107,800 USD for flexible pavement and 70,792 USD for rigid pavement were calculated. The sustainability analysis based on CO_2_ emissions during initial construction demonstrated that the use of GGBFS reduced CO_2_ emissions by 11.95% in flexible pavements and 7.50% in rigid pavements. It was evaluated that the largest source of these emissions is the material production process, which accounts for approximately 57% of total emissions in flexible structures and approximately 95% in rigid structures.

## 1. Introduction

Today, developing engineering structures in a safe, economical, and sustainable manner is among the primary objectives of engineering practices. The longevity of these structures, which are designed particularly on variable terrain conditions, climatic circumstances, and diverse soil formations, depends largely on the engineering properties of the underlying soil, such as strength, permeability, and consolidation. Roads constructed on weak and problematic soils that fail to meet design standards are subjected to structural issues over time, such as loss of bearing capacity, settlement, and surface de-formation [1]. These issues not only increase financial risks but also jeopardize traffic safety. In cases where relocating the project site is technically or economically unfeasible, it is of paramount importance to resort to soil improvement methods to ensure structural continuity and minimize maintenance costs [2,3]. In improvement processes, mechanical methods based on regulating the grain size distribution of soils or chemical techniques utilizing pozzolanic additives such as lime, cement, and fly ash stand out [4]. In recent studies, industrial wastes such as blast furnace slag, fly ash, and rice husk ash have also begun to find a place in soil reinforcement processes. However, in recent years, the use of industrial and waste materials in soil improvement has been steadily increasing due to concerns regarding environmental sustainability and economic efficiency [5,6,7,8,9,10,11,12].

Previous studies on soil stabilization have demonstrated the positive effects of various binders and industrial waste materials on mechanical properties. It is observed that rice husk ash, lime, cement, fly ash, and various industrial by-products are widely used in soil improvement. Rahgozar et al. [5] conducted unconfined compression tests to improve clay soils using cement and rice husk ash, determining that the addition of 4% cement and 10% rice husk ash was the optimum mixture ratio. In a study by Yadav et al. [6] aimed at improving a clay soil with rice husk ash and sugarcane bagasse ash, it was stated that the addition of 7.5% rice husk ash increased the bearing capacity, whereas the same effect was not observed with sugarcane bagasse or cow dung ash. Reddy et al. [7] tested soil stabilization using fly ash, metakaolin, and their combinations. The experimental results showed that the highest strength values were obtained with the addition of 9% fly ash, 12% metakaolin, and a combination of 8% fly ash + 6% metakaolin. Mesfun et al. [8] conducted various experiments to investigate the stabilization of expansive clayey road sub-grade using lime and pumice powder. As a result of the CBR tests, they determined that the highest strength was achieved in the mixture containing 7% lime + 3% pumice powder. Perera et al. [9] performed a series of mechanical and microstructural analyses by adding crushed glass to clay soils at different ratios (0%, 5%, 10%, 15%, and 20%). According to the results, they found that the addition of 10–15% crushed glass significantly improved mechanical properties such as unconfined compressive strength, CBR, and modulus of elasticity, while reducing swelling and shrinkage potentials and increasing the water-holding capacity of the clay. Harikaran et al. [10] researched the stabilization of clayey soil using lime and waste eggshell powder. They obtained the highest strength values with a combination of 9% lime and 12% waste eggshell powder. Bharti et al. [11], in their study on the stabilization of black cotton soil with copper slag, prepared samples by mixing copper slag at four different ratios (5.0%, 10.0%, 15.0%, and 20.0%) and applied modified Proctor, CBR, unconfined compression, liquid limit, and plastic limit tests on the mixtures. Their findings indicated that the optimum mixture was achieved at a 20% copper slag ratio. Wassie and Demir [12] found that in stabilization experiments conducted with cement and metakaolin on inorganic silt of high plasticity, the best unconfined compression values were reached with the addition of 8% cement and 3% metakaolin, and a significant increase occurred in shear strength parameters. Furthermore, there is a growing momentum in geotechnical studies, which constitute the core subject of soil improvement [13,14]. In addition to conventional industrial by-products, recent studies have increasingly explored novel eco-friendly binders for soil and aggregate base stabilization. For instance, waste glass powder-based geopolymers and recycled aggregates have emerged as promising materials to enhance the mechanical, microstructural, and durability properties of aggregate bases and clayey subgrade soils [15,16,17,18,19]. Furthermore, alternative industrial residues such as lowly-reactive coal gasification fly ash (CGFA) and various coal ash derivatives have also been successfully utilized to stabilize aggregate bases and subgrade layers, demonstrating their viability as sustainable geotechnical construction materials [20,21,22,23].

Blast furnace slag, an industrial waste and a byproduct of the iron and steel manufacturing process, contains significant amounts of silica, alumina, and calcium oxide in its structure. It is well known that this material, obtained as a byproduct during iron and steel production, acquires hydraulic binding properties after the grinding stage and develops pozzolanic reactions upon contact with soil. Numerous studies in the literature have confirmed that, owing to these characteristic features, the material in question can be used as an effective additive in the stabilization of various soil types [24,25,26,27,28,29]. In a study by Abdila et al. [24], a significant increase was observed in the CBR values of soil samples reinforced with GGBFS; mechanical compaction characteristics were determined by establishing the optimum moisture content and dry unit weight parameters based on standard Proctor tests. Uzer et al. [25] stated that geopolymers based on blast furnace slag and fly ash offer a sustainable solution for the stabilization of clayey soils, emphasizing that modeling performed using Artificial Neural Networks (ANN) statistically confirmed the increase in soil strength. Hu et al. [26] proved that combinations of blast furnace slag and fly ash, when analyzed through machine learning-supported optimization techniques, provide ideal strength and deformation characteristics for flexible pavement designs. The setting behavior and mechanical performance of slag-based geopolymers are also influenced by their chemical composition, particularly the role of calcium, which can affect reaction processes, setting time, and strength development [27,28]. Liu and Hung [29] reviewed recent advances in sustainable soil stabilization and reported that industrial by-products, particularly GGBFS, are widely utilized to improve the hydromechanical and mechanical properties of soils through cementation, pore-filling, and hydration mechanisms, while simultaneously reducing the environmental impacts associated with conventional stabilizers. In recent years, with the increasing emphasis on environmental sustainability, various waste and recycled materials have been utilized in soil stabilization. Sustainability aims to meet the needs of the present without compromising the ability of future generations to meet their own Federal Highway Administration [30], and the assessment and mitigation of the environmental impacts of engineering practices are of great importance in this regard. In this context, reducing greenhouse gas emissions, particularly CO_2_, which accounts for 95% of total emissions, remains one of the primary objectives [31].

In this context, carbon footprint research focusing on the analysis of greenhouse gas emissions plays a critical role in evaluating the sustainability of highway design and construction activities within the scope of transportation [32,33,34,35,36,37] comparatively examined crushed stone and lime-stabilized soil layers used in highway projects in terms of greenhouse gas emissions. Previous life-cycle assessment (LCA) studies have consistently demonstrated that material production is the dominant contributor to greenhouse gas emissions in pavement systems, frequently accounting for more than half of the total environmental burden during the construction stage. Consequently, replacing conventional construction materials with industrial by-products such as GGBFS has emerged as an effective strategy for reducing embodied carbon while simultaneously improving pavement performance [38,39]. Zhang et al. [34] reported a 57.09% reduction in emissions as a result of using lime-stabilized soils and geogrid-reinforced embankments. Kaewunruen et al. [35] analyzed the carbon footprint of the Beijing–Shanghai high-speed railway line, identifying that the construction phase accounted for the largest share of total emissions at 64.86%, and emphasized the importance of implementing eco-friendly technologies. Giustozzi et al. [36] recommended the preference of stabilization with cement by demonstrat-ing its potential to reduce emissions by over 80%. Gupta et al. [37] reduced CO_2_ emissions per kilometer from 910.9 tons to 750.8 tons through the use of stabilization additives in a four-lane road section.

Globally, approximately 300 kg of slag is generated for every ton of raw iron produced, resulting in the production of approximately 390 million tons of blast furnace slag annually [40]. Several studies have reported that replacing conventional cementitious materials with ground granulated blast furnace slag (GGBFS) substantially decreases greenhouse gas emissions because GGBFS is an industrial by-product requiring considerably lower additional processing energy than Portland cement [41,42]. Previous life-cycle assessment studies have consistently shown that material production is the dominant contributor to the total carbon footprint of pavement systems, frequently accounting for more than half of total emissions in flexible pavements and an even greater proportion in concrete pavements. The reduction of greenhouse gas emissions during pavement construction has become one of the primary objectives of sustainable transportation infrastructure, and the selection of low-carbon materials is considered one of the most effective mitigation strategies [38,39]. Due to the quantity and environmental impact of the blast furnace slag generated each year, investigating its utilization for disposal is considered of great importance from the perspective of sustainability and the conservation of natural resources.

Despite the extensive body of research on blast furnace slag-based soil stabilization, limited studies have comprehensively integrated mechanical performance evaluation with sustainability assessment through carbon footprint analysis within a unified experimental framework. In particular, the combined investigation of subgrade strength improvement, pavement thickness optimization based on AASHTO design methodology, and CO_2_ emission comparison for flexible and rigid pavements remains insufficiently addressed in the literature.

In this context, the present study investigates the stabilization of bentonite soil with GGBFS for use as a highway subgrade material. Mechanical performance was evaluated through laboratory experiments, and pavement design implications were assessed using the AASHTO 1993 method. Furthermore, a sustainability-based approach was adopted by calculating construction-phase CO_2_ emissions, enabling a comparative evaluation of environmental and economic performance. Although several studies have investigated soil stabilization using GGBFS [15,16,17,18,19,20,43,44,45], an integrated approach that directly compares the simultaneous impacts of unactivated GGBFS on flexible and rigid pavement designs (AASHTO 1993), unit-area initial construction costs, and stage-specific construction CO_2_ emissions remains limited. This study fills this gap in the literature by offering a holistic framework that directly links the mechanical performance enhancements of optimum GGBFS addition to field-scale pavement thickness optimization, unit cost savings, and CO_2_ emission reductions.

## 2. Materials and Methods

### 2.1. Materials

BT, evaluated as the road subgrade material, was obtained from ÇANBENSAN Çankırı Bentonit San. Tic. A.Ş. (Çankırı, Türkiye) and is characterized by its low shear strength, high compressibility, high plasticity, and significant swelling potential. These properties can lead to a reduction in bearing capacity and structural failures due to swelling-shrinkage behavior. The primary mineral component of bentonite is montmorillonite. The properties of the samples, prepared in accordance with the relevant standards [46,47,48,49], were determined. The soil is classified as CH, with a bulk density of 0.9 g/cm^3^, an optimum moisture content of 35.25%, a maximum dry unit weight of 1.310 g/cm^3^, a measured liquid limit (LL) of 398.2%, a plastic limit (PL) of 48.6%, and a plasticity index (PI) of 349.6%. Furthermore, the percentage of material retained on a 150 μm sieve was determined to be 2.50%, while the percentage retained on a 75 μm sieve was 12.5%. Upon examining the chemical composition of the material, the proportions were determined as follows: 2.41% Na_2_O, 2.68% MgO, 16.54% Al_2_O_3_, 63.40% SiO_2_, 0.13% P_2_O_5_, 0.41% SO_3_, 0.03% Cl, 1.10% K_2_O, 6.91% CaCO_3_, 0.76% TiO_2_, 0.02% V_2_O_5_ and Cr_2_O_3_, 0.08% MnO, and 5.51% Fe_2_O_3_.

**Figure 1:**
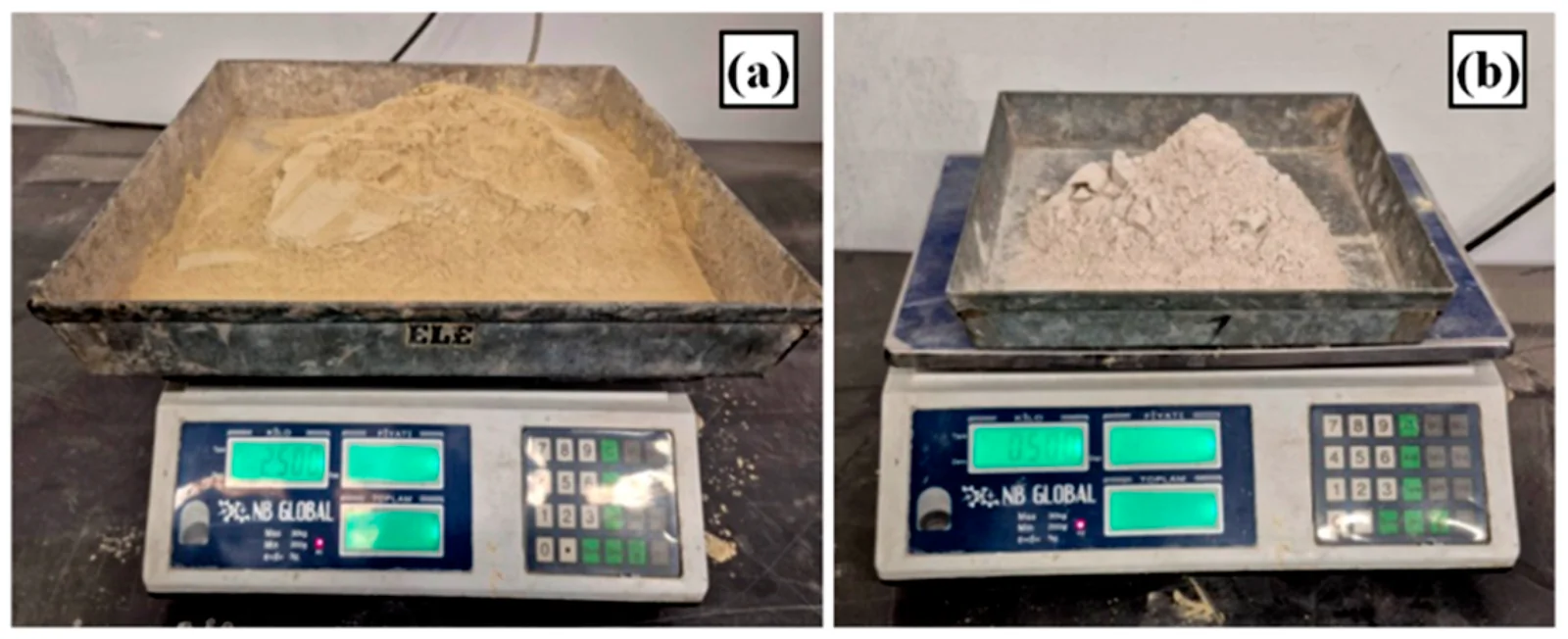
Bentonite (**a**) and GGBFS (**b**) samples.

GGBFS is an industrial byproduct with hydraulic binding properties, obtained by rapidly cooling molten slag from blast furnace with water during the iron-steel production process to convert it into granular form, followed by grinding to micron sizes. Due to the high content of CaO, Al_2_O_3_, and SiO_2_ in its composition, it exhibits binding characteristics by developing pozzolanic reactions. This chemical structure allows GGBFS to be used as an eco-friendly binder in geopolymer production and soil stabilization applications. Studies in the literature demonstrate that the use of GGBFS improves engineering properties while contributing to the reduction of the carbon footprint [25,43]. The GGBFS used in this study was obtained from ISDEMIR (Iskenderun Iron and Steel Inc., Hatay, Türkiye). When evaluating the chemical composition of the material, the proportions were identified as 33.6% CaO, 41.2% SiO_2_, 14.3% Al_2_O_3_, 7.3% MgO, 0.8% S, 0.8% MnO, 0.9% K_2_O, and 1.1% TiO_2_. Figure 1a shows the bentonite sample, and Figure 1b shows the GGBFS sample.

### 2.2. Methods

#### 2.2.1. Preparation of Samples

After determining the physical and chemical properties of the soil sample (BT), the optimum moisture content (w_opt_) and maximum dry unit weight (γ_dmax_) values were established using the standard Proctor test (ASTM D698) [47]. Compaction was executed in a standard 101.6 mm (4 in.) diameter mold in three equal layers, applying 25 blows per layer using a 2.5 kg (5.5 lb) rammer dropped from a height of 305 mm (12 in.), delivering a standard compaction energy of 600 kN-m/m^3^. Mixtures containing 5%, 10%, 15%, and 20% GGBFS by dry weight of soil (added as an additive based on dry soil mass) were prepared. Each mixture was compacted at its respective w_opt_ and γ_dmax_ parameters to maintain consistent density control. Unconfined compressive strength (UCS) specimens were prepared (ASTM D2166) [50] using a cylindrical metallic mold with dimensions of 38 mm in diameter and 76 mm in height (height-to-diameter ratio of 2:1) via dynamic compaction. Following compaction, UCS specimens were wrapped tightly in plastic sheets and stored in airtight containers to prevent moisture loss, cured at room temperature (23 ± 2 °C) for curing periods of 3, 7, and 28 days prior to testing. Additionally, California Bearing Ratio (CBR) specimens were prepared in accordance with ASTM D1883 [40] in a standard 152.4 mm (6 in.) diameter mold with a spacer disc, compacted in three equal layers with 56 blows per layer using a standard Proctor rammer(UTEST Material Testing Equipment, Ankara, Türkiye). To prevent moisture loss and eliminate environmental exposure, the CBR specimens were wrapped in plastic sheets and sealed in airtight containers, cured at a constant room temperature of 23 ± 2 °C for 3, 7, and 28 days. Immediately following the completion of the specified curing periods, CBR testing was performed directly without unsoaked/soaked conditioning (unsoaked/direct testing after curing) to evaluate the bearing capacity and load-penetration behavior of the stabilized soils. For each mixture ratio and curing condition, at least three replicate specimens were prepared and tested to ensure data repeatability and experimental accuracy.

#### 2.2.2. Experimental Method

**Figure 2:**
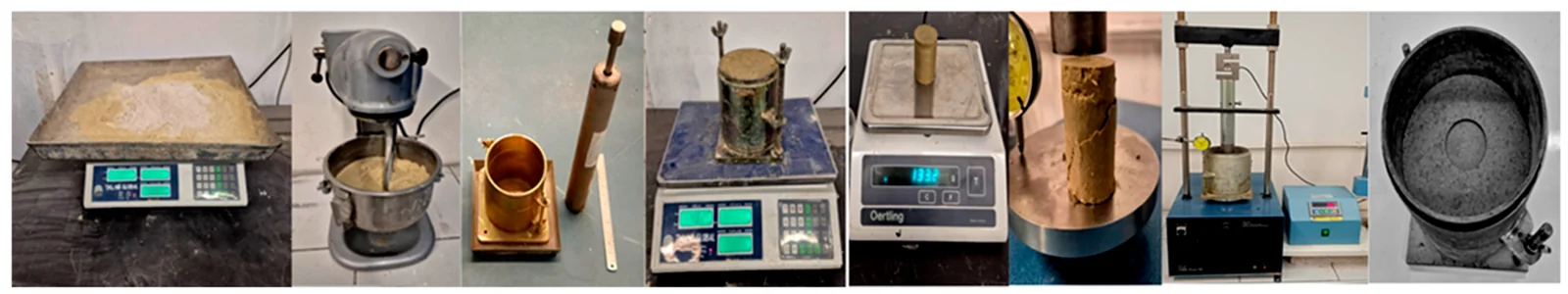
Standard proctor test, unconfined compression test, CBR test applications.

The standard Proctor test (ASTM D698) is applied to determine the maximum dry unit weight and optimum moisture content of a soil sample [47]. The unconfined compression test (ASTM D2166) measures the uniaxial compressive strength of soil samples [50]. The California Bearing Ratio (CBR) test (ASTM D1883) evaluates the bearing capacity of the soil by analyzing the load-penetration relationship [51]. The applications of these tests are shown in Figure 2. The chemical properties of the materials and mixtures, as well as the determination of surface morphology, were analyzed using scanning electron microscopy (SEM) [29].

#### 2.2.3. Design

A highway structure consists of subgrade (the shaped road cross-section) and pavement (subbase, base, and surface layers) components built to withstand traffic loads. Design parameters such as soil properties, traffic density, regional conditions, and cost play a decisive role in determining layer thicknesses and the service life of the project [52]. Within the framework of this research, both flexible and rigid pavement designs are considered.

**Figure 3:**
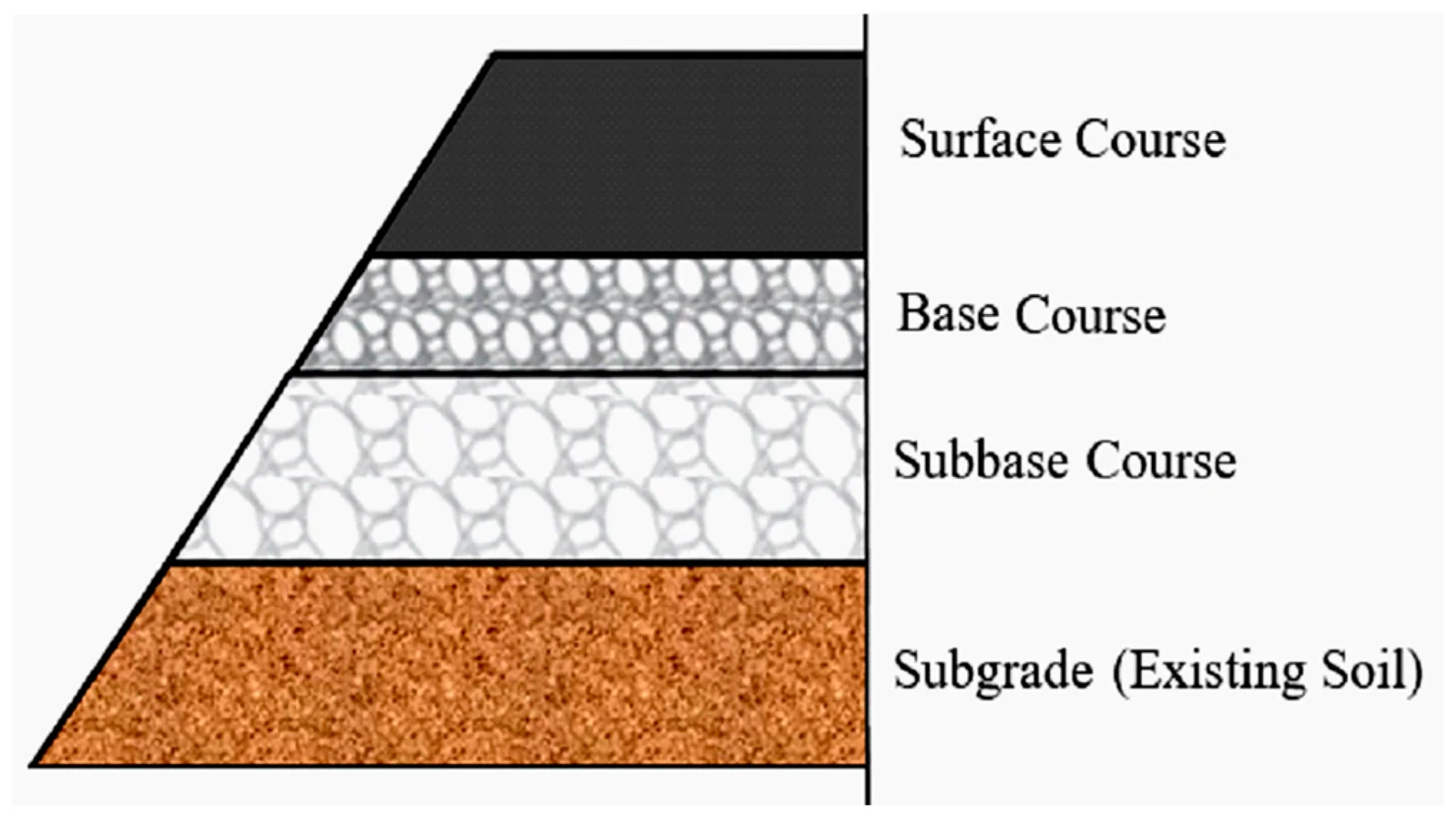
Flexible pavement sectional view.

Flexible pavements, which utilize asphalt cement as a binding agent, are designed according to the AASHTO [52] method; this system typically consists of surfacing, base, and subbase layers. The surfacing layer, containing bitumen and aggregate, is composed of the wearing course, binder course, and bituminous base [53]. Figure 3 illustrates the layering in a flexible road structure.

In determining the layer thicknesses, Equation (1), provided in accordance with the AASHTO [52] Design Guide, is utilized [52].

(1) $$\log\;(W_{8.2})=Z_{R}\;\times \;S_{0}+9.36\;\times \;\log(\mathrm{SN}+1)\;-\;0.20\frac{log(\frac{∆PSI}{4.2-1.5})}{0.40+\frac{1094}{{\left(SN+1\right)}^{5.19}}}+2.32\times \log\;M_{R}-8.07$$

Here, W_8.2_ represents the 8.2 tons equivalent standard single axle load repetition (project traffic), MR is the resilient modulus of subgrade soil (psi), S_0_ is the combined standard error of traffic and performance forecast, Z_R_ is the standard normal deviation, SN is the structural number (inches), and ∆PSI is the difference between initial (P_o_) and final service (P_t_) capability index. After the Structural Number (SN) is obtained through Equation (1), the thicknesses of the layers forming the flexible pavement are calculated via Equation (2), taking the layer coefficients into account [52].

SN = a_1_ × D_1_ + a_2_ × D_2_ × M_2_ + a_3_ × D_3_ × M_3_………….ai × Di (2)

**Figure 4:**
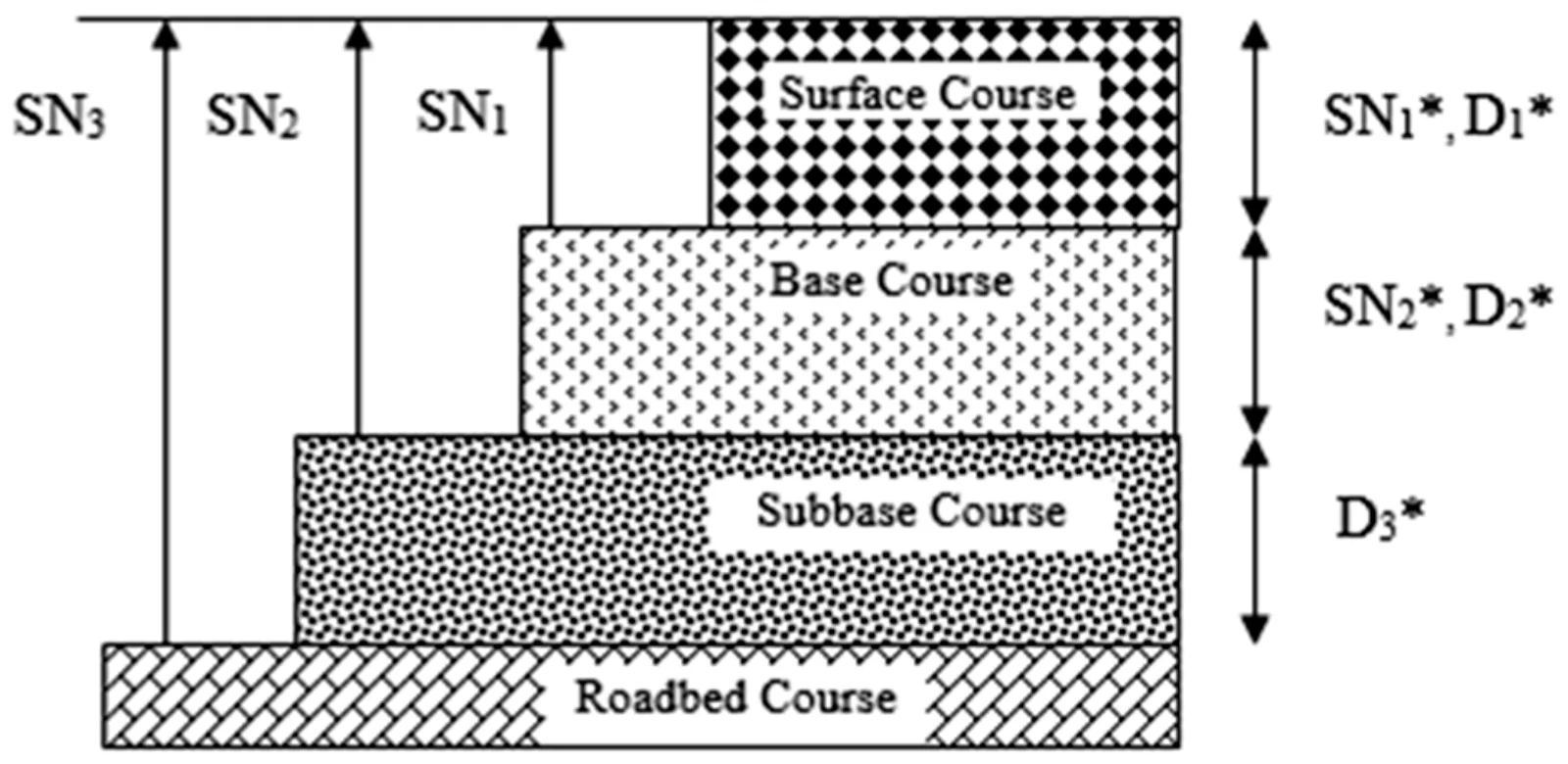
Pavement numbers in flexible pavement [52]. (where SNi represents the required structural numbers, SNi* represents the provided structural numbers, and Di* represents the provided layer thicknesses).

In the aforementioned formula, the “D” letters symbolize the layer thicknesses, while the “a” letters represent the strength coefficients of the layers. In the calculations, the base and subbase drainage coefficients (M_2_, M_3_) were assumed to be unity (1.00), and these values were obtained from the AASHTO 1993 guide. The adequacy of the determined flexible pavement thicknesses was verified through the SN* calculations specified in Figure 4.

**Figure 5:**
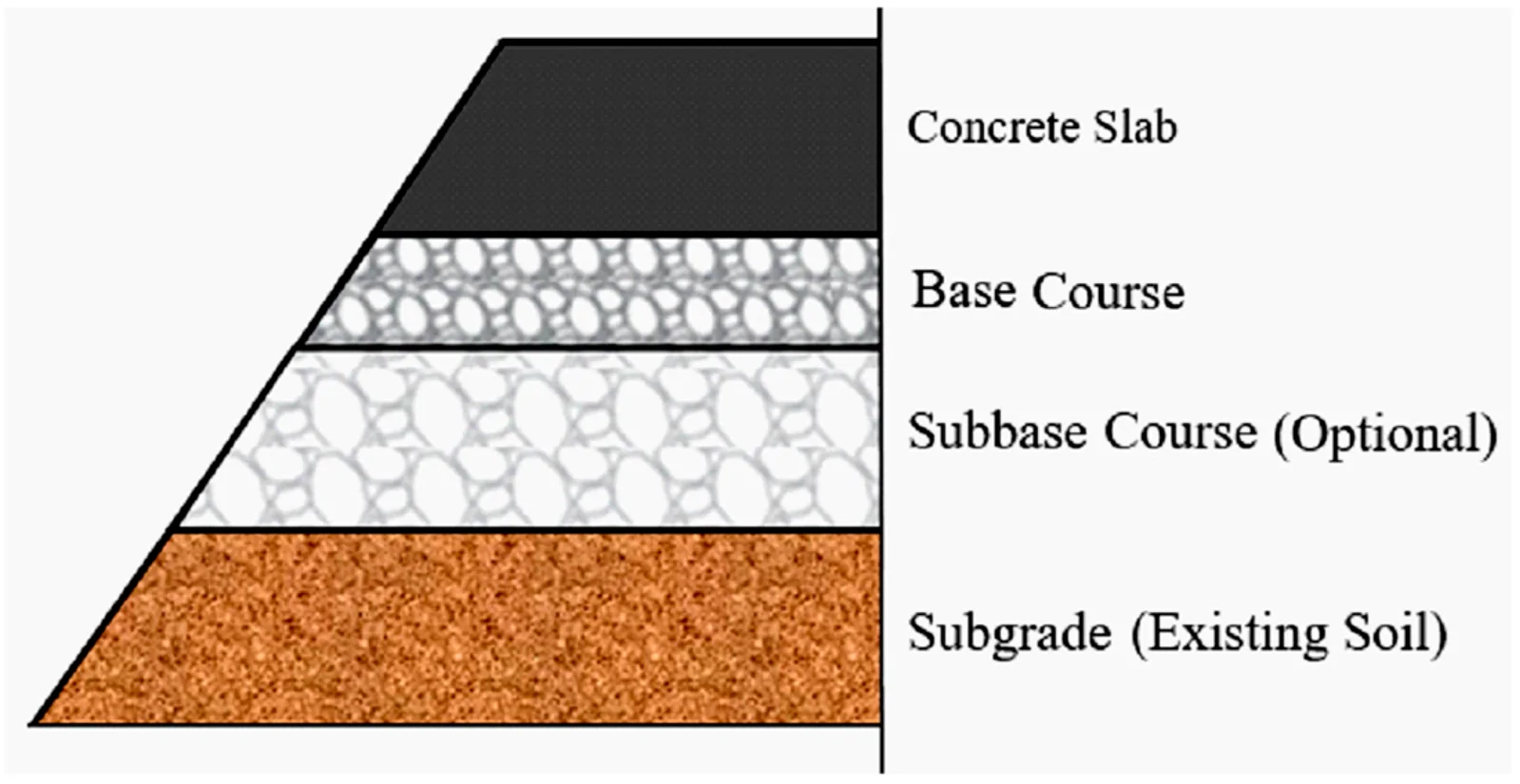
Rigid pavement section view.

Under the AASHTO [41] framework for rigid pavement design, a Portland cement concrete slab is constructed directly upon the subgrade. Precise determination of the concrete slab and constituent layer thicknesses is vital to endure deformations induced by heavy traffic loads. The sectional configuration and layering of the rigid pavement are illustrated in Figure 5.

According to AASHTO [41] guidelines, layer thicknesses are calculated using Equation (3) [52].

(3) Log10(w8,2)=ZR. S0+7.35.log10 (d+1) − 0.06+log.10[∆PSI4.2−1.5]1+1.624.107(d+1)8.46+(4.22−0.32.Pt).log10 [S′c.Cd(d0.75−1.132)215.63.J[d0.7518.42(Ec/k)0.25]]

In this context; W_8.2_ denotes the cumulative 8.2-ton equivalent single axle load (ESAL) repetitions for the project traffic, while ZR and So represent the standard normal deviation and the overall standard error for traffic and performance predictions, respectively. The variable d indicates the concrete slab thickness (in inches), and ΔPSI, signifies the loss in serviceability between the initial P_o_) and terminal (P_t_) indices. Other parameters include the concrete modulus of rupture (S′_c_), the load transfer coefficient (J), the drainage coefficient (C_d_), the elastic modulus of concrete (E_c_), and the modulus of subgrade reaction (k). This design incorporated an optimized stabilizer blend to enhance structural integrity and assess its influence on the rigid pavement system. Furthermore, the analysis adhered to the standards for Jointed Plain Concrete Pavement (JPCP) in accordance with the Turkish Highways Concrete Pavement Design Guidelines [54].

#### 2.2.4. Sustainability Analysis Based on CO_2_ Emissions During Initial Construction

This research performed a comparative analysis between flexible and rigid pavement designs, utilizing CBR values derived from both untreated and GGBFS stabilized soils. Design thicknesses for the various layers were established in accordance with the AASHTO 1993 methodology. For a divided highway section measuring 1000 m × 20 m, the carbon footprint was quantified, accounting for the stabilized layer as well. The sustainability assessment was centered on the initial construction phase, evaluating CO_2_ emissions associated with material manufacturing, logistics, and the operation of construction machinery.

In flexible pavements, Hot Mix Asphalt (HMA) is composed of 93–97% aggregate and 3–7% bitumen, with the bitumen serving as the binding agent [53]. This study quantifies CO_2_ emissions from HMA production by adopting emission factors of 190 kg/ton for bitumen [55] and 6.5 kg/ton for aggregate [36], while neglecting the relatively insignificant emissions associated with water usage (0.3 kg/ton) reported by Giustozzi et al. [36]. The unit weight of the asphalt material is taken as 2.4 tons/m^3^. Conversely, rigid pavements consist of a Portland cement concrete slab laid over a granular base. Cement remains a primary source of carbon emissions, accounting for 7–8% of global total emissions, with clinker production being the major contributor. To mitigate this, mineral additives industrial by-products with zero CO_2_ emissions are utilized as cement replacements. For the purposes of this study, C35/45 grade concrete was selected in accordance with Turkish standards, featuring an estimated emission intensity of 339.57 kg/m^3^ [56].

GGBFS is obtained through the rapid water quenching of slag, a byproduct of iron and steel manufacturing. Since GGBFS is an industrial byproduct, it does not involve primary raw material extraction, as similarly emphasized in the literature [57]. However, to avoid overestimating environmental benefits and maintain analytical transparency, energy consumed during post-processing, drying, and fine grinding stages was taken into account. Following relevant Life Cycle Assessment (LCA) literature and ISO 14040/14044 principles, an embodied carbon emission factor of 0.035 kg CO_2_/kg (35 kg CO_2_/ton) was assigned to GGBFS in this study to account for processing activities [58,59,60,61], while its logistical impacts were separately calculated under transportation emissions. Transportation logistics were modeled using two operational radii: a 50 km local radius for local aggregates, asphalt mixtures, and ready-mixed concrete, and a 500 km regional haulage radius for GGBFS supplied from centralized industrial steel complexes (e.g., Iskenderun Iron and Steel Works) via national highway corridors.

This study adopted the IPCC Tier 1 methodological approach [62,63] to calculate total CO_2_ emissions resulting from road construction materials (bitumen, aggregate, concrete) and the stabilizer (GGBFS) for a representative road section. Total material-related embodied emissions were quantified using Equation (4), based on CO_2_ emission factors per unit mass (kg/ton) or volume (kg/m^3^)

Eco_2_ = DA × FE (4)

Here; E_CO2_ = Total CO_2_ emission (kg CO_2_), DA = Activity Data representing the total material quantity supplied for the road construction, expressed in terms of mass (ton) for stabilization and flexible pavement layers or volume (m^3^) for rigid pavement concrete slabs, and FE = Specific CO_2_ Emission Factor per unit mass (kg CO_2_/ton) or unit volume (kg CO_2_/m^3^) [62,63].

For mass-based calculations (flexible pavement components and GGBFS-stabilized subgrade), the Activity Data (DA) in tons is calculated as: DA = l × b × h × γ

Where l = length of the road (m), b = width of the road (m), h = layer thickness (m), and γ = compacted dry density/unit weight of the road material (ton/m^3^). For volume-based calculations (rigid pavement concrete slab), DA is calculated directly as DA = l × b × h (m^3^).

To provide a clear and reproducible calculation example, consider a subgrade layer stabilized with an illustrative 10% additive content (by dry weight of soil) for a 1000 m long and 20 m wide road section with a stabilized thickness of 0.15 m:

Total layer volume = 1000 m × 20 m × 0.15 m = 3000 m^3^.

Total mass of stabilized subgrade layer (assuming a compacted dry density = 1.80 ton/m^3^) = 3000 m^3^ × 1.80 ton/m^3^ = 5400 tons.

Mass of stabilizing additive required (DA at 10% replacement ratio) = 5400 tons × 0.10 = 540 tons.

Total CO_2_ emission from stabilizer production (assuming an illustrative embodied emission factor FE = 100 kg CO_2_/ton) = 540 tons × 100 kg CO_2_/ton = 54,000 kg CO_2_ (54 tons CO_2_).

This study calculated the CO_2_ emissions resulting from the construction of a flexible pavement comprising wearing, binder, bituminous base, granular base, subbase, and GGBFS-stabilized road subgrade layers. The analysis encompassed material production, transportation (utilizing 10-ton trucks over a distance of 50 km), and on-site operations such as laying, compaction, and spraying. Specific equipment and procedures were considered for each layer. Transportation emissions were calculated using the value of 2.96 kg CO_2_/ton [37], and total emissions were determined via Equation (4). Furthermore, the study evaluated the CO_2_ emissions of rigid pavement construction (concrete slabs) and the GGBFS-stabilized road subgrade, focusing on transportation, machinery, and equipment usage. For concrete transportation, 6.5 m^3^ transit mixers (9.5 kg CO_2_/m^3^, 50 km) were used [57,64,65], while 10-ton trucks (2.96 kg CO_2_/ton, 50 km) were preferred for stabilizer transportation [37,64]. On-site operations such as laying, compaction, and curing were included in the analysis (Equation (5)). Machinery emissions were calculated using fuel consumption data from the KGM in Turkey and a modified equation (Equation (6)); the constructions of standard and GGBFS-stabilized road subgrades were compared. Equation (4) was utilized for transportation calculations.

*F* = *BSFC. P. T.* 1/*γ* (5)

ε_CO2_ = *F*. *α* (6)

Here; F = Fuel Consumption (L), BSFC = Brake specific fuel consumption (g/(kW.h)), *p* = Motor power at the rotational speed that provides the maximum torque (kW), T = Time (hour), γ = Density of fuel used (0.832 kg/L). CO_2_ = Total CO_2_ emission (kg), α = CO_2_ emitted when consuming 1 L of diesel (2.6639 kg/L) [36].

## 3. Results

This study investigated the effects of GGBFS-stabilized road subgrade on highway pavements and its sustainability based on CO_2_ emissions generated during initial construction. Standard Proctor and unconfined compression tests were performed on untreated bentonite soil and GGBFS-reinforced samples following curing periods of 3, 7, and 28 days. CBR tests were conducted on samples with an optimum GGBFS content of 15%. Using the CBR results, flexible and rigid pavement layer thicknesses were designed according to the AASHTO 1993 method, and pavement costs were calculated. Sustainability analyses based on CO_2_ emissions were performed for both the untreated bentonite soil and GGBFS-stabilized designs.

### 3.1. Standard Proctor and Unconfined Compression Test Results

**Figure 6:**
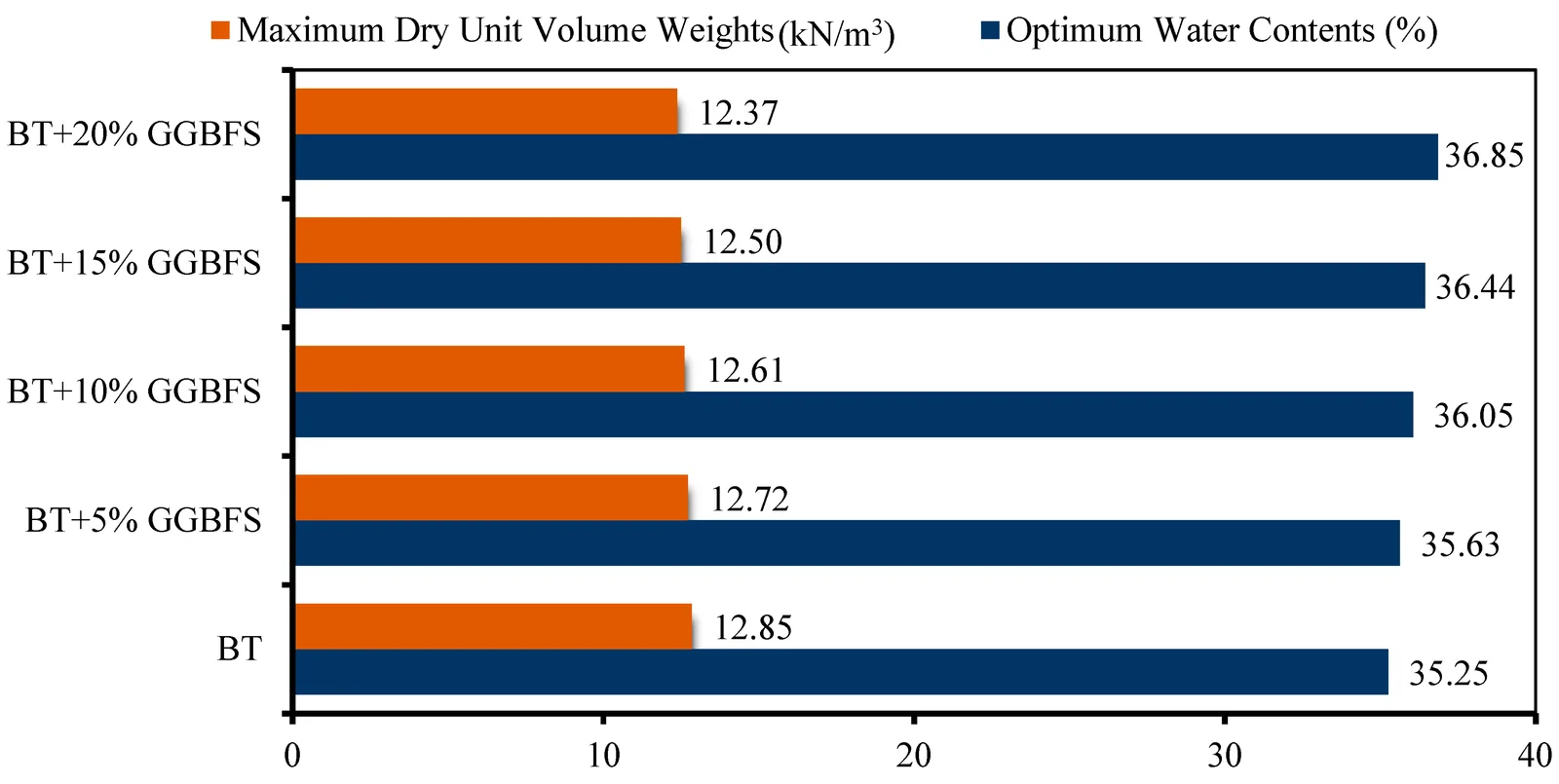
Standard proctor test results.

**Figure 7:**
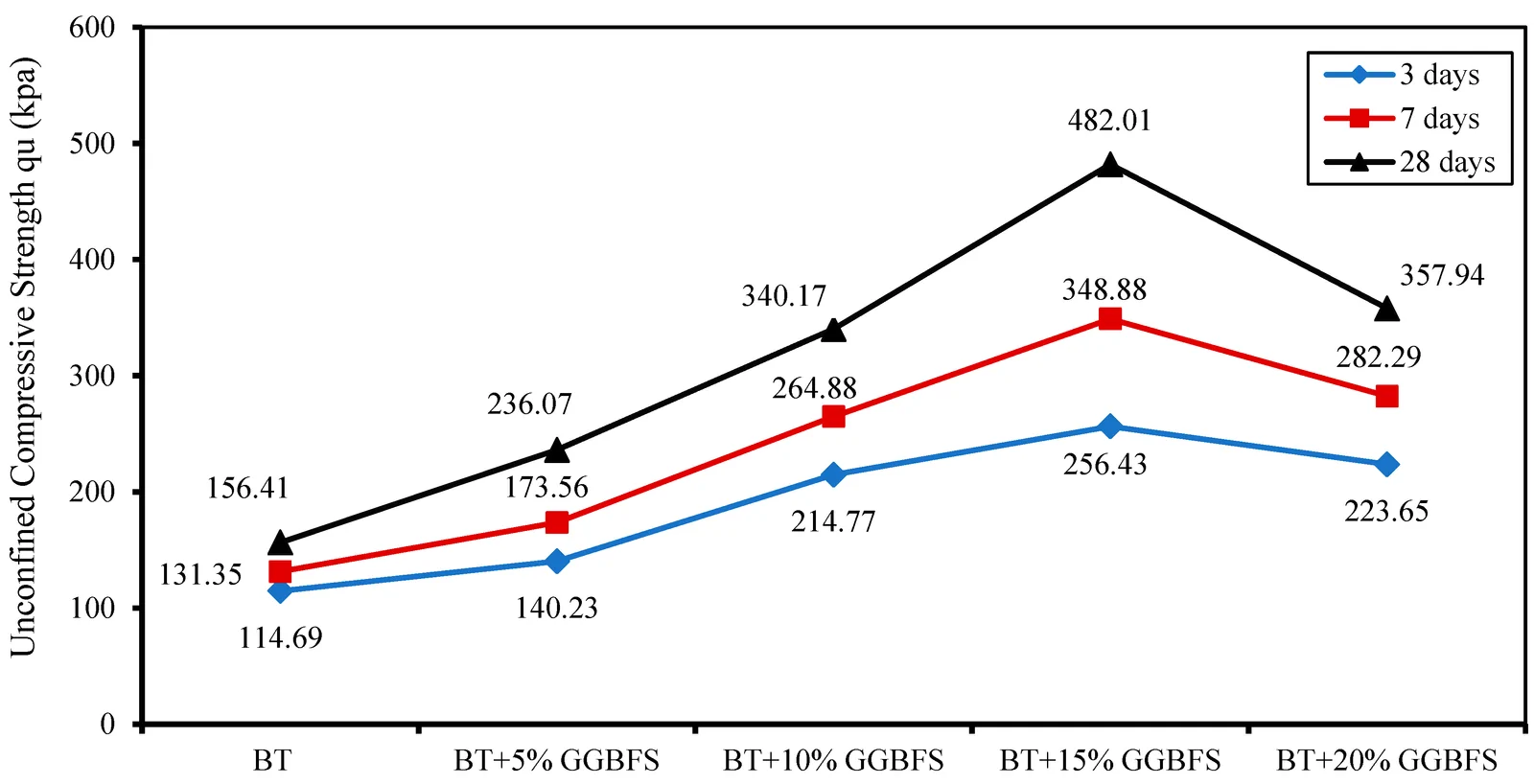
Change of UCS values depending on the additive ratio and curing period.

**Figure 8:**
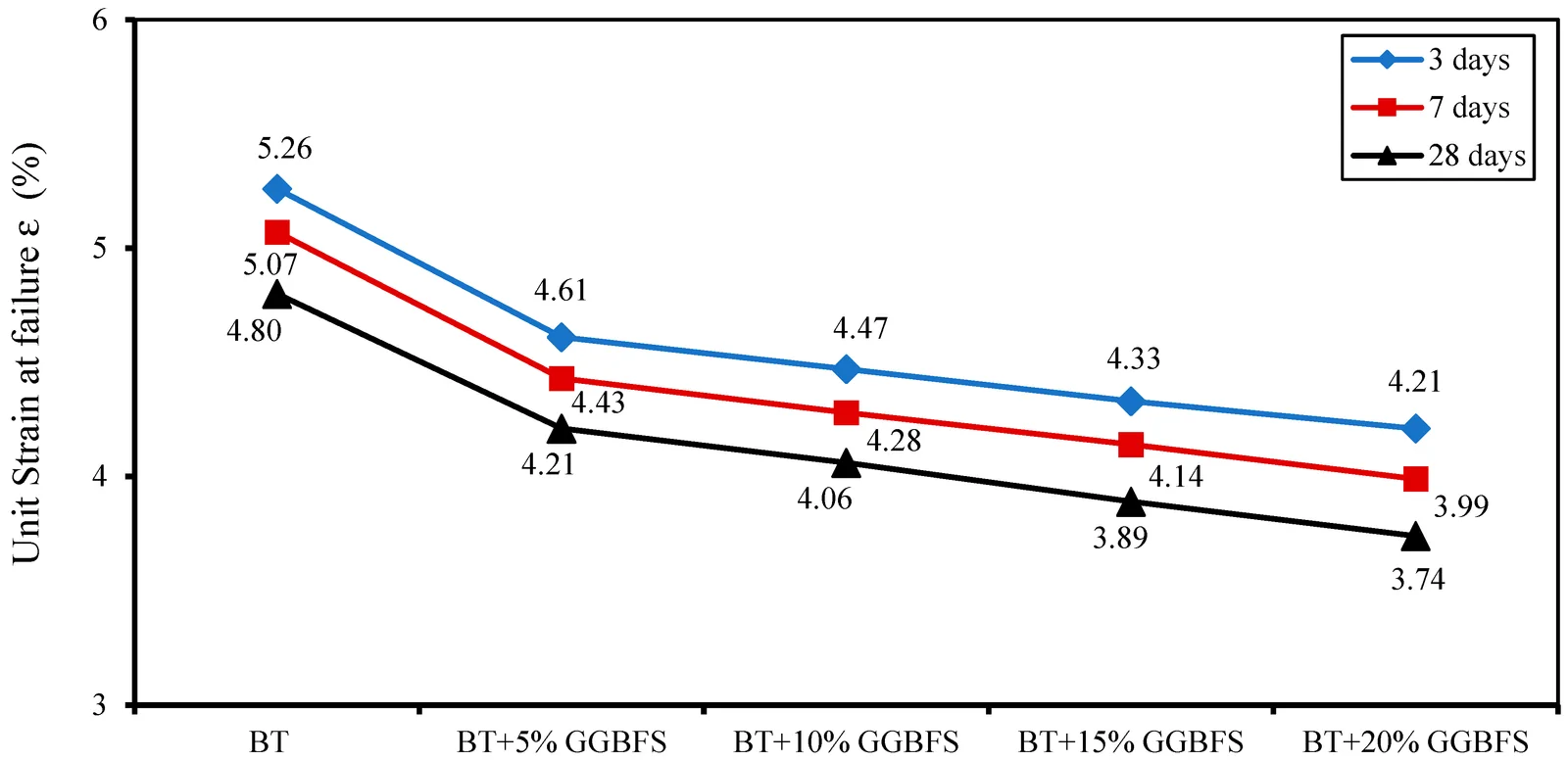
Change of unit strain depending on additive ratio and curing period.

Standard Proctor tests [47] were performed on untreated bentonite soil and soil samples with GGBFS additives at ratios of 5%, 10%, 15%, and 20%. It was found that the increase in GGBFS substitution led to an increase in the w_opt_ values while causing a decrease in the γ_dmax_ values. These obtained data are in parallel with the existing literature [44,45,66,67]. This trend is evaluated to result from the fact that GGBFS increases the void ratio of the mixture due to its low specific gravity and porous texture, thereby increasing water demand by making compaction more difficult. This situation is observed to overlap with similar studies in the literature [45,67,68]. Unconfined compression tests were applied to the soil samples prepared using the w_opt_ and γ_dmax_ values. The samples were tested following curing periods of 3, 7, and 28 days. The experimental results are shown in Figure 6, Figure 7 and Figure 8.

Unconfined compression tests demonstrated that increasing GGBFS concentration and curing periods enhanced the compressive strength of the soil. Samples containing 15% GGBFS exhibited the highest strength; at the end of 3, 7, and 28 days, increases of 124%, 166%, and 208% were recorded, respectively, compared to the untreated bentonite soil. This positive effect of GGBFS additive on strength is in parallel with studies in the literature [45,66]. Furthermore, the decrease observed in strain values, parallel to the increase in GGBFS content and curing period, is associated with the increase in the material’s stiffness, and these results overlap with the data in the literature [69].

### 3.2. CBR Test Results

Samples prepared within the scope of the CBR test were based on untreated BT soil and the optimal mixture ratio of 15% GGBFS identified from the unconfined compression test results. All test specimens were compacted at their corresponding maximum dry unit weight (γ_dmax_) and optimum moisture content (w_opt_) parameters using standard Proctor compaction energy in accordance with ASTM D1883. To prevent moisture loss and maintain controlled curing conditions, the compacted specimens were wrapped tightly in plastic sheets, sealed in airtight containers, and stored in a temperature-controlled environment (23 ± 2 °C) for curing periods of 3, 7, and 28 days. Following the completion of each specified curing period, unsoaked direct CBR tests were performed. To ensure statistical reliability and experimental repeatability, all testing was conducted on three replicate specimens per mix formulation, and the absolute CBR values were expressed as mean ± standard deviation (SD).

**Figure 9:**
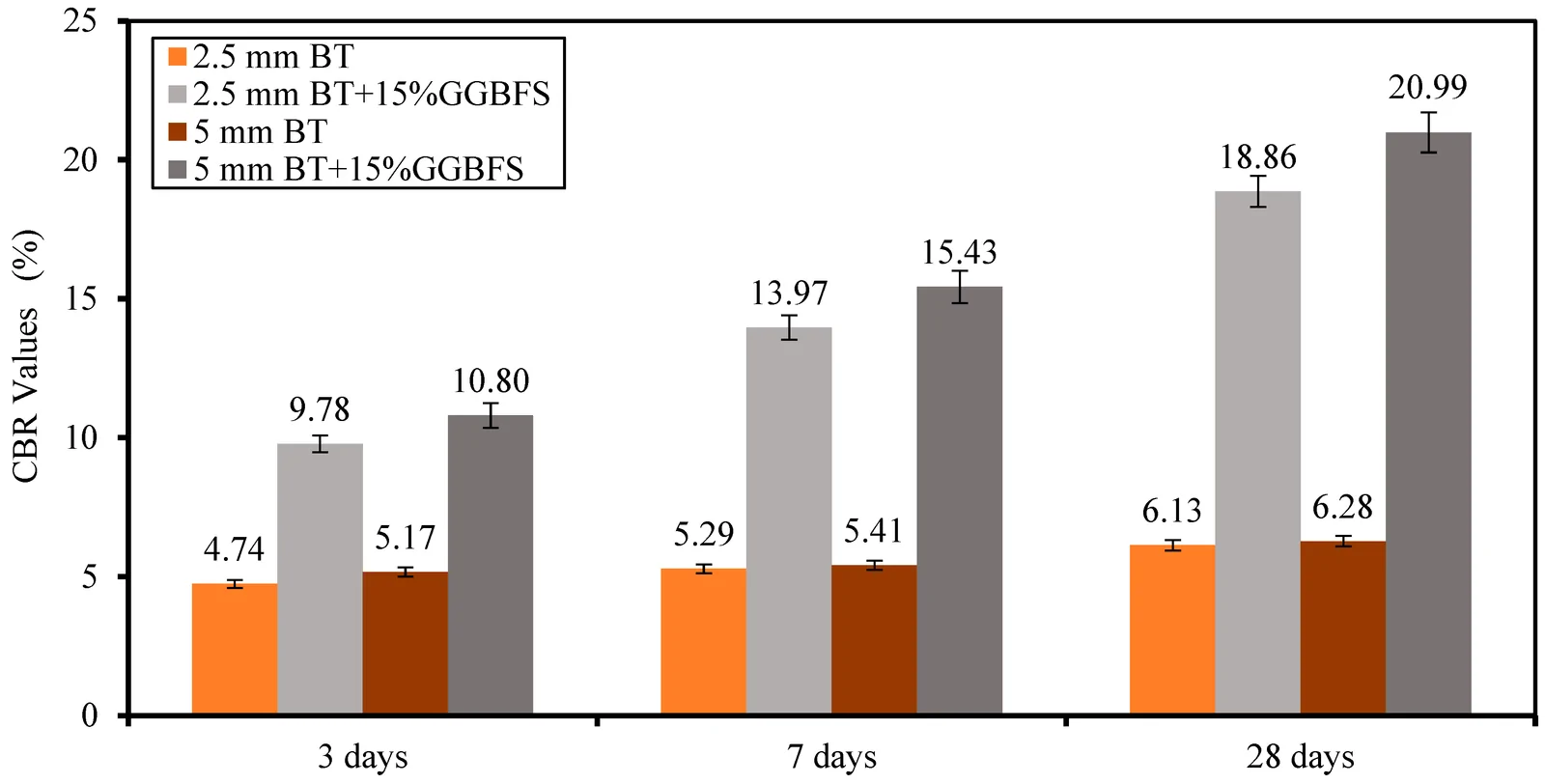
Change of CBR values depending on curing time.

The detailed CBR test results, including individual specimen measurements, mean values, and standard deviations, are presented in Figure 9. In accordance with AASHTO standards, the CBR values evaluated at 5.0 mm penetration depth were adopted as the primary structural design benchmark for subgrade resilient modulus conversions and pavement thickness calculations. Upon examining the CBR test results, it was determined that GGBFS stabilization significantly increased the bearing capacity of the soil. For the optimal 15% GGBFS-stabilized soil, the CBR values exhibited significant increases for 2.5 and 5 mm penetrations, respectively: 106% and 109% after a 3-day curing period (reaching 9.78%, SD = 0.30% and 10.80%, SD = 0.45%); 164% and 185% after a 7-day curing period (reaching 13.97%, SD = 0.44% and 15.43%, SD = 0.58%); and 208% and 234% after a 28-day curing period (reaching 18.86%, SD = 0.56% and 20.99%, SD = 0.72%), compared to the untreated BT specimens (which recorded 4.74%, SD = 0.14% and 5.17%, SD = 0.16% at 3 days; 5.29%, SD = 0.16% and 5.41%, SD = 0.16% at 7 days; and 6.13%, SD = 0.19% and 6.28%, SD = 0.19% at 28 days for 2.5 and 5 mm penetrations, respectively). The low standard deviation values across all test groups demonstrate high experimental consistency and data repeatability.

The observed increase in the CBR values of the GGBFS-added mixtures compared to the untreated sample is in parallel with literature findings [25,66,67] indicating that the use of GGBFS enhances soil bearing capacity. Research in the literature shows that the reactive nature of calcium, silica, and alumina in the GGBFS content increases mechanical resistance by forming hydration products. It has been identified that GGBFS, which exhibits both hydraulic and pozzolanic properties, is particularly significant in improving clay soils with low bearing capacity and high plasticity. While our current experimental findings confirm that the use of GGBFS improves mechanical performance, they also reveal that exceeding the optimum value leads to the accumulation of unreacted free material and therefore does not create an additional increase in strength.

### 3.3. Microstructural Analysis Results

**Figure 10:**
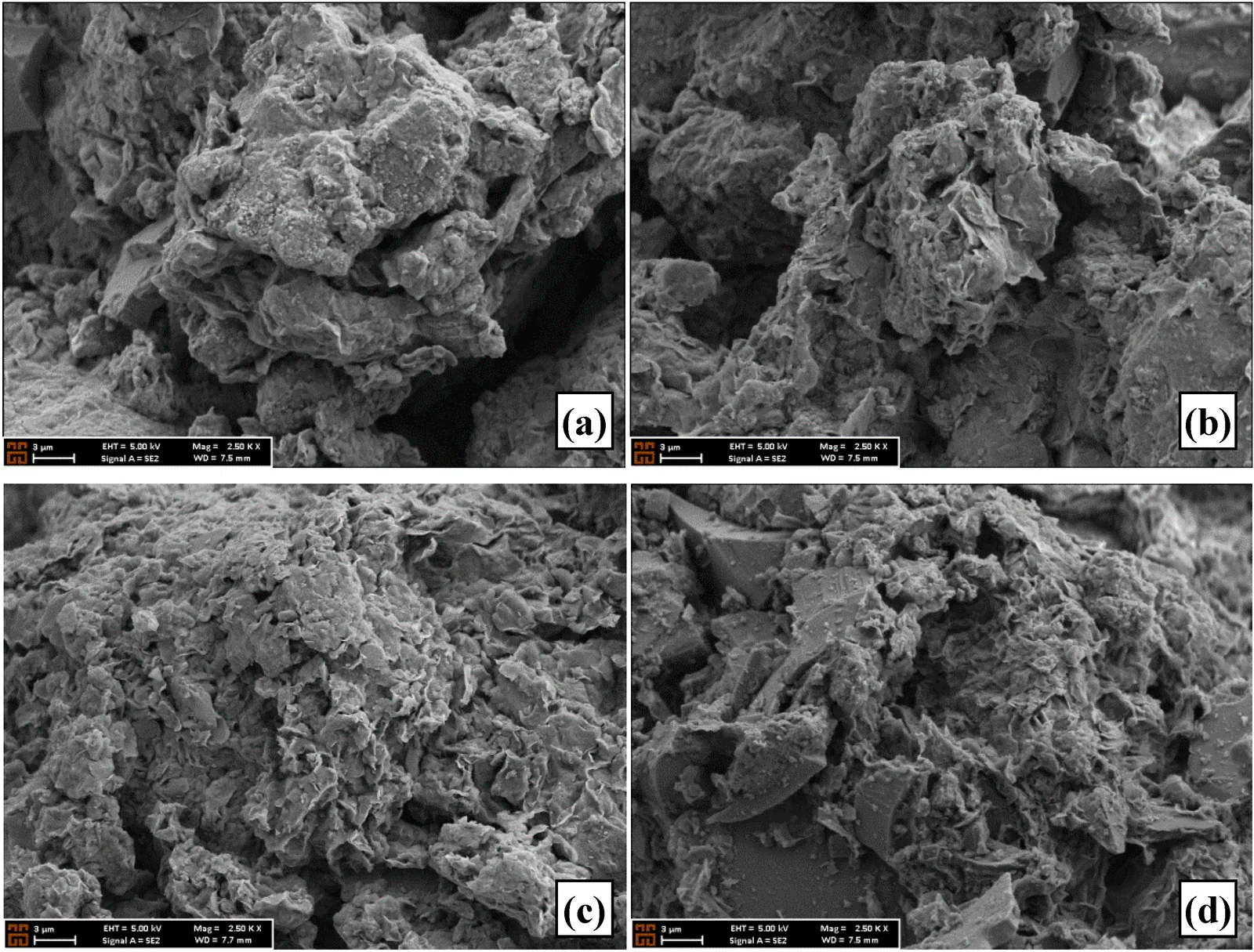
SEM images of additive soil samples; (**a**) 5% GGBFS, (**b**) 10% GGBFS, (**c**) 15% GGBFS, (**d**) 20% GGBFS.

As a result of the tests conducted in this study, the optimum mixture ratio was determined to be 15% GGBFS, based on tests performed by adding 5%, 10%, 15%, and 20% GGBFS to untreated BT soil samples. SEM images, prepared to observe the effect of GGBFS in mixtures prepared at different ratios and to explain the stabilization mechanism of the improvement, are provided in Figure 10.

The strength development and the identification of 15% GGBFS as the optimum dosage can be attributed to the synergistic combination of cation exchange, particle agglomeration, and pozzolanic reaction mechanisms occurring without the addition of any chemical activators (unactivated GGBFS system). Upon mixing bentonite with GGBFS in the presence of water, Ca^2+^ ions released from the calcium-rich GGBFS (33.6% CaO) induce cation exchange on the montmorillonite clay surfaces, reducing the double-layer thickness and promoting flocculation. Subsequently, long-term pozzolanic reactions occur between the dissolved silica and alumina species derived from both the highly plastic bentonite (LL = 398.2%) and the GGBFS (which provides 41.2% SiO_2_ and 14.3% Al_2_O_3_), resulting in the formation of matrix-filling cementing hydration products, microstructurally consistent with calcium silicate hydrate (C-S-H) and calcium aluminate hydrate (C-A-H) gel phases reported in the literature [25,70,71]. As evidenced by the SEM morphology in Figure 10c, maximum gel formation and a highly compact, dense microstructure were achieved at the 15% GGBFS content. Conversely, increasing the GGBFS dosage to 20% did not yield further strength gain and resulted in a performance plateau. In this unactivated system, where no external alkaline activator (such as NaOH or Na_2_SiO_3_) was introduced, the inherent alkalinity of the pore solution was insufficient to fully dissolve and react the excess GGBFS particles. Consequently, as illustrated in Figure 10d, unreacted GGBFS grains remained embedded within the clay matrix. These unreacted particles acted as non-binding inclusions and weak interfacial zones within the soil structure, preventing further mechanical improvement and confirming 15% as the chemical saturation threshold for the unactivated bentonite-GGBFS system.

### 3.4. Durability Performance (Freeze-Thaw Resistance)

**Figure 11:**
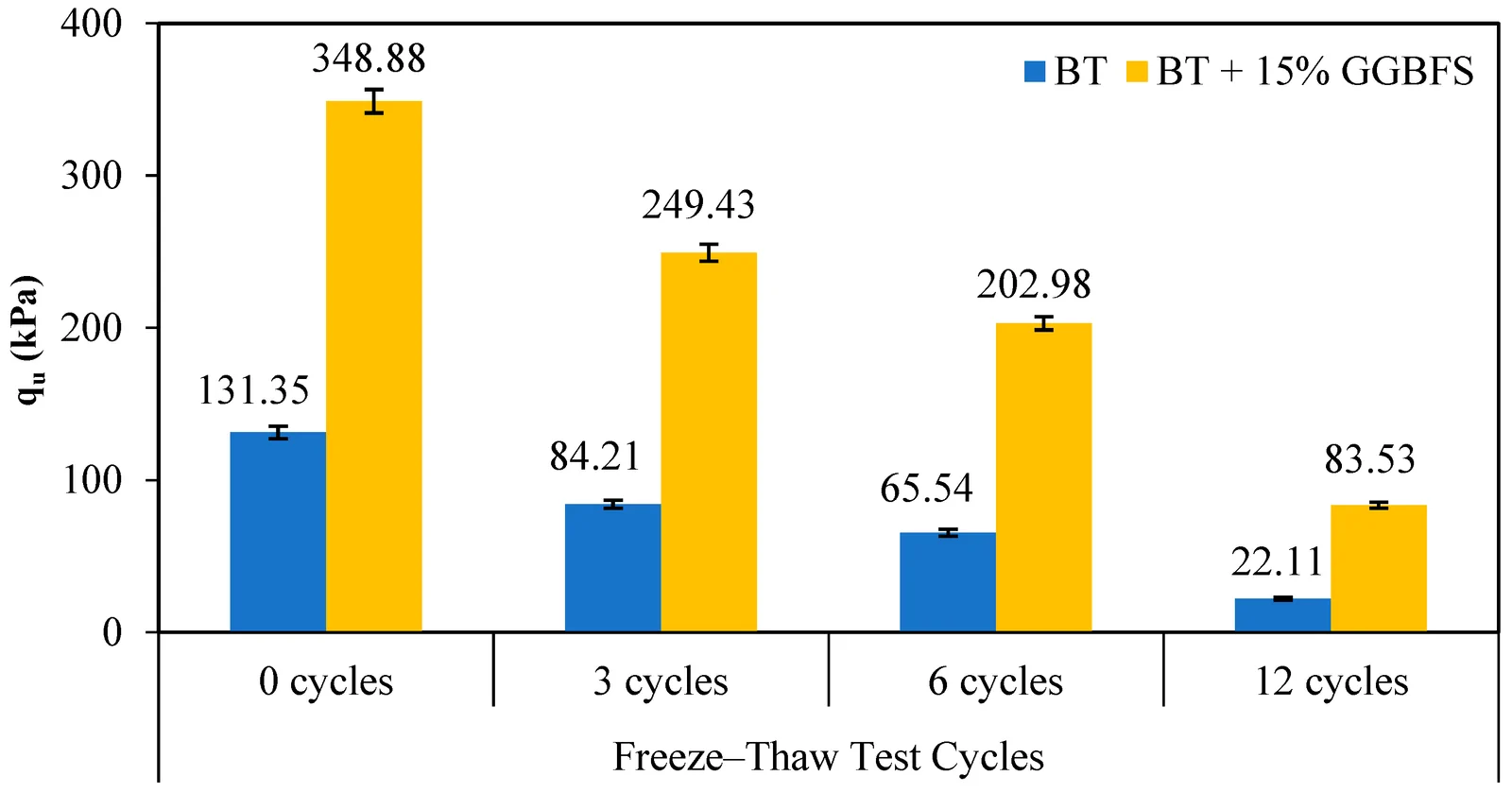
Unconfined compressive strength (qu) variations and error bars (mean ± SD, n = 3) of untreated BT soil and 15% GGBFS-stabilized soil across 0, 3, 6, and 12 freeze-thaw cycles.

**Figure 12:**
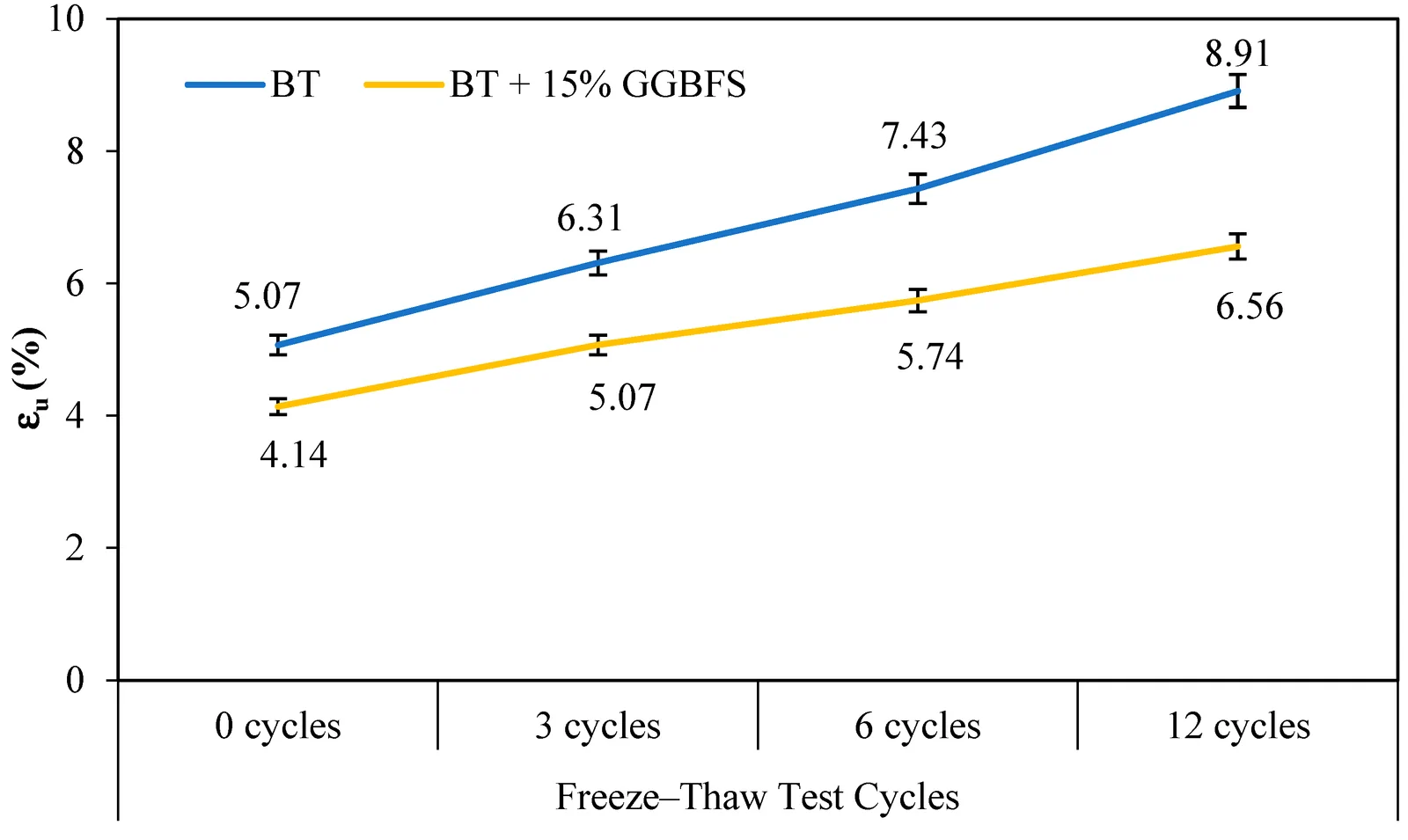
Axial strain at failure (ε_u_) variations and error bars (mean ± SD, n = 3) of untreated BT soil and 15% GGBFS-stabilized soil across 0, 3, 6, and 12 freeze-thaw cycles.

The freeze-thaw durability test is conducted to assess the detrimental microstructural deterioration caused by recurring phase transitions of pore fluid, which expands upon freezing at sub-zero conditions and melts as ambient temperatures rise above zero. This environmental durability assessment was carried out in strict accordance with the testing protocols outlined in the ASTM D560/D560M-16 [72] standard. The specimen preparation and compaction procedures established for the unconfined compressive strength (UCS) tests were utilized as the foundation for fabricating the durability samples. Following an initial 7-day curing process, the test specimens were placed in a climatic freeze-thaw chamber and exposed to freezing conditions at −23 degrees C for 24 h, followed by a thawing phase at a regulated room temperature of 23 ± 2 °C for 23 h. This consecutive freezing and thawing sequence defines one full thermal cycle. Upon achieving the specified cyclic intervals, the post-exposure strength retention and physical integrity of the specimens were determined to complete the testing process [72,73]. The cycle limits were configured to satisfy both the minimum requirement of 5 cycles specified in the Turkish General Directorate of Highways (KGM) technical guidelines [73] and the comprehensive 12-cycle benchmark mandated by ASTM D560/D560M-16 [72]. For comparative performance evaluations, the baseline unconfined compressive strength (0 cycles) determined after 7 days of curing for the optimal mixture design was assigned as the control reference. Upon concluding the designated cyclic exposures, the resulting mass variations and percentage losses in unconfined compressive strength were systematically documented and analyzed (Figure 11 and Figure 12 and Table 1).

Upon evaluating the freeze-thaw test results across 3, 6, and 12 cycles presented in Figure 11 and Figure 12, and Table 1, the unconfined compressive strength (qu) values for untreated BT and 15% GGBFS-stabilized specimens were recorded as 84.21 kPa and 249.43 kPa at 3 cycles; 65.54 kPa and 202.98 kPa at 6 cycles; and 22.11 kPa and 83.53 kPa at 12 cycles, respectively. The standard deviation (SD) values for qu in untreated BT specimens were 2.78 kPa, 2.30 kPa, and 1.05 kPa for 3, 6, and 12 cycles, whereas the corresponding SD values for the 15% GGBFS mixture were 5.60 kPa, 4.40 kPa, and 2.07 kPa, respectively. Regarding the axial failure strain (eu) parameters, the values for untreated BT and 15% GGBFS-stabilized samples were determined as 6.31% and 5.07% at 3 cycles; 7.43% and 5.74% at 6 cycles; and 8.91% and 6.56% at 12 cycles, respectively. The corresponding strain SD ranges were evaluated as 0.18%, 0.22%, and 0.25% for untreated BT, and 0.15%, 0.17%, and 0.19% for 15% GGBFS-blended samples across the same cyclic intervals. These narrow standard deviation boundaries confirm strong structural reproducibility and experimental consistency under cyclic thermal stress [74].

Relative to baseline 0-cycle values, untreated BT specimens exhibited strength and weight losses of 35.89% and 2.78% at 3 cycles; 50.10% and 3.23% at 6 cycles; and 73.74% and 3.86% at 12 cycles. In contrast, 15% GGBFS-added specimens demonstrated significantly reduced strength and weight losses of 28.51% and 1.39% at 3 cycles; 41.82% and 1.67% at 6 cycles; and 66.51% and 2.03% at 12 cycles, respectively (Table 1 ). While initial strength improvement was 166% at 0 cycles, the relative performance advantage of the stabilized matrix expanded to 196%, 210%, and 278% after 3, 6, and 12 freeze-thaw cycles, underscoring enhanced durability.

The superior freeze-thaw resistance and restricted physical detachment of soil aggregates in GGBFS-stabilized specimens are attributed to the dense microstructural network created by pozzolanic hydration products [74,75,76]. The formation of calcium silicate hydrate (C-S-H) gels fills micro-voids, reduces hydraulic conductivity, and inhibits capillary water migration that otherwise feeds ice lens growth in highly plastic clays during freezing phases [74,77,78]. Additionally, the high SiO_2_ content inherent to GGBFS plays a key role in reinforcing the soil skeleton against cyclic freeze-thaw degradation [74,79].

### 3.5. Pavement Design

In this study, it was assumed for the pavement design that the subgrade consists of untreated BT soil and BT + 15% GGBFS samples. Flexible and rigid pavement designs were carried out in accordance with the AASHTO 1993 design guide, using the highest CBR values obtained at the end of 3, 7, and 28-day curing periods (5.17%, 5.41%, and 6.28% for untreated BT soil; 10.80%, 15.43%, and 20.99% for 15% GGBFS-added soil, respectively). In the calculations, the resilient modulus (MR) of the subgrade was first determined. For the MR estimation, the correlation between MR and CBR values presented in the 3rd edition of the “Mechanistic-Empirical Pavement Design Guide” (MEPDG-3), published by AASHTO in 2024 [80] and including mechanistic-empirical technical revisions, was utilized. This correlation is expressed as MR (psi) = 2555 (CBR)^0.64^ [80] and was used to determine the MR values. In addition, the modulus of subgrade reaction (k) was calculated using the relationship k = MR/19.4 [52]. The selection of these empirical correlations is justified by their widespread recognition in AASHTO design standards, offering a practical and standardized basis for preliminary design-level pavement evaluations. Nevertheless, it is recognized as a study limitation that chemical stabilization using GGBFS significantly alters the soil matrix, introducing stress-dependent, non-linear deformation characteristics that may differ from untreated soils. While direct cyclic triaxial testing for MR determination provides superior precision for detailed site-specific designs, the utilization of CBR-based empirical correlations remains a widely accepted, standardized, and practical approach supported by AASHTO guidelines for comparative preliminary design evaluations under time and economic constraints. Furthermore, all design input parameters and structural coefficients were selected in accordance with the Turkish General Directorate of Highways specifications (KGM 2008 Flexible Pavement Design Guide and KGM 2019 Rigid Pavement Design Guide) [53,54] to reflect heavy traffic design conditions. The resulting structural layer thicknesses were also checked against KGM minimum layer thickness requirements to ensure practical constructability in the field. Furthermore, the subgrade stabilization cost was evaluated as 0.85 USD/m^2^. Considering that GGBFS is an industrial by-product, this unit cost strictly incorporates the 500 km maximum haulage logistics of GGBFS using the official KGM transport formulation, alongside the standard equipment and labor unit rates recommended by KGM for in-situ soil mixing, watering, and compaction operations (specifically tractor ripper scarification, bulldozer levelling, motor grader mixing, water tanker spraying, vibratory roller compaction, and pneumatic-tired roller finishing). A sensitivity evaluation of the cradle-to-gate inventory indicates that raw material manufacturing—specifically bitumen production for flexible pavements (~57% of total emissions) and Portland cement production for rigid pavements (>95% of total emissions)—overwhelmingly dominates the overall environmental footprint. Consequently, even when evaluating extended GGBFS transport distances (up to 500 km), the substantial reduction in pavement layer thickness achieved via 15% GGBFS subgrade stabilization provides a decisive, robust net reduction in cumulative CO2 emissions across both pavement types.

### 3.6. Flexible Pavement Design

The M_R_ values calculated for the untreated BT soil were 7312, 7527, and 8281 psi (50.4, 51.9, and 57.1 MPa, respectively) according to the respective 3, 7, and 28-day curing periods; for the 15% GGBFS-added soil, these values were calculated as 11,716, 14,271, and 17,926 psi (80.8, 98.4, and 123.6 MPa, respectively). Total pavement layer thicknesses were calculated by incorporating the general values in Table 2 and the resilient modulus values into Equation (1). To represent heavy traffic corridor conditions and highlight the maximum potential of subgrade stabilization under critical loading scenarios, a design traffic volume of 200,000,000 ESALs was adopted in alignment with KGM (2008) specifications. Structural layer coefficients (a_1_ = 0.42 for asphalt wearing, a_2_ = 0.40 for binder, a_3_ = 0.36 for bituminous base, a_4_ = 0.15 for plant-mix base, and a_5_ = 0.13 for subbase) and drainage coefficients (m = 1.0) recommended in KGM (2008) [53] were utilized to distribute the required Structural Number (SN) across individual pavement layers, while adhering to minimum practical layer thickness thresholds.

The impact of the GGBFS-stabilized soil on flexible highway pavement costs was evaluated in accordance with the data provided by the KGM. In the unit price schedules published by KGM, unit volume (m^3^ or ton) costs are presented for subbase and base layers, while unit area (m^2^) costs are provided for bituminous base, binder, and wearing courses. In this study, the 2026 KGM unit prices [81] presented in Table 3 were used for the economic analysis of the highway pavements.

**Figure 13:**
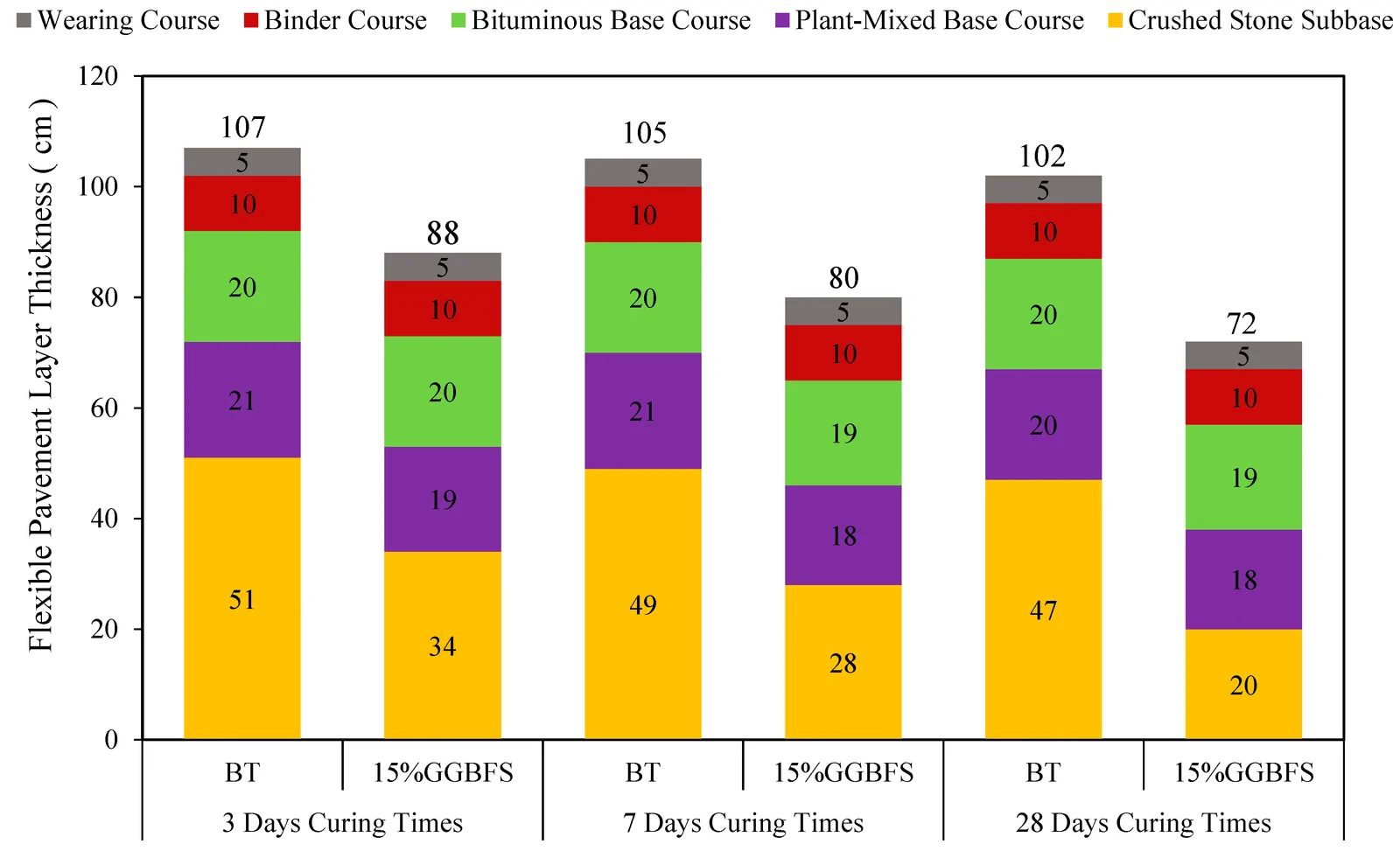
Flexible pavement layer thicknesses of BT and GGBFS stabilized soils.

**Figure 14:**
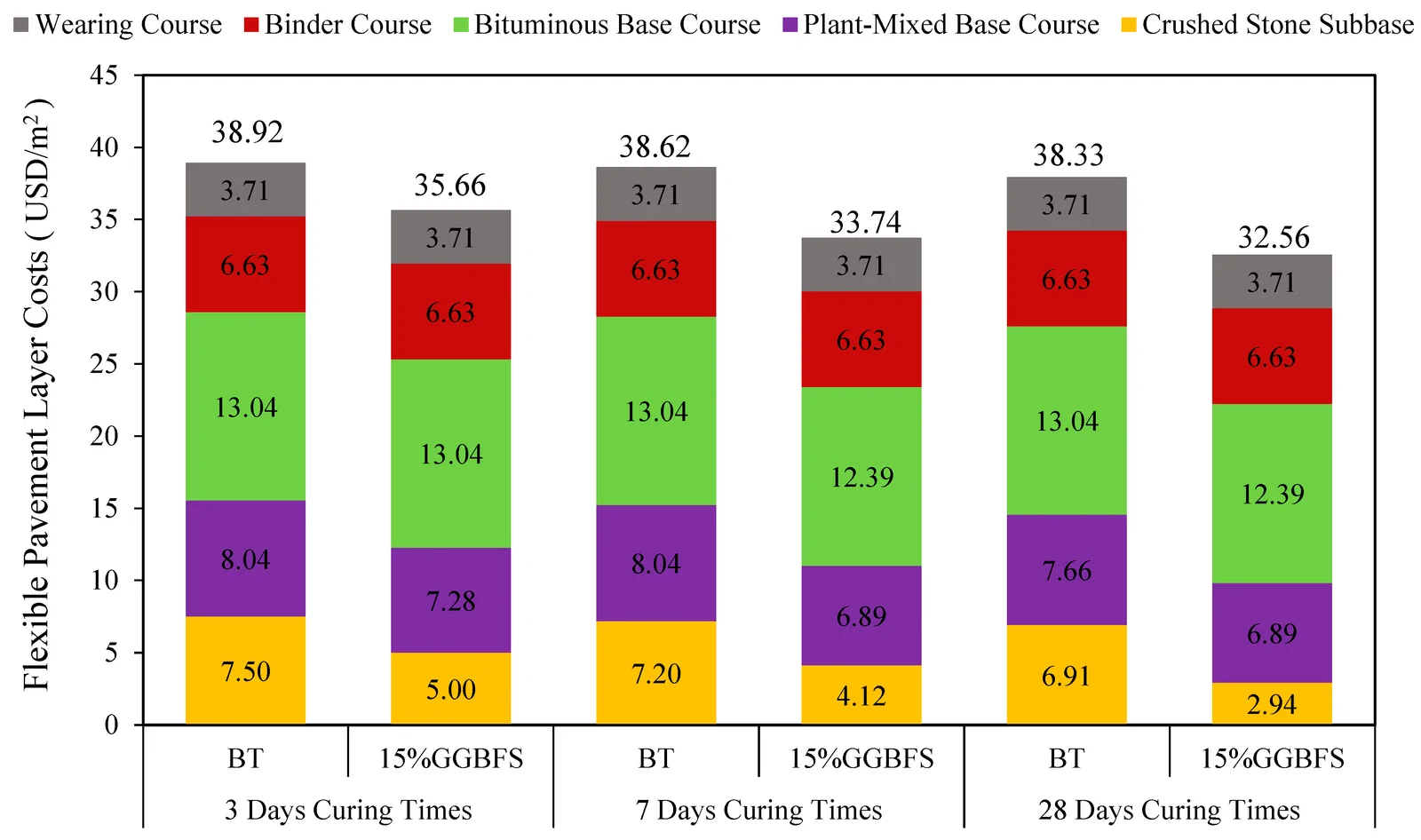
Flexible pavement layer costs calculated for of BT and GGBFS stabilized soils.

Figure 13 and Figure 14 demonstrate that, compared to the untreated BT soil, the layer thicknesses of the GGBFS-stabilized soil decreased by 17.76%, 23.81%, and 29.41% for 3, 7, and 28-day curing periods, respectively. The construction costs of flexible pavement using 15% GGBFS-stabilized soil decreased by 8.38%, 12.64%, and 14.20% per unit area (m^2^) compared to the untreated BT soil, depending on the curing periods. For a divided highway with dimensions of 1000 m × 20 m, this corresponds to savings of 65,200 USD, 97,600 USD, and 107,800 USD for the 3, 7, and 28-day curing periods, respectively.

### 3.7. Rigid Pavement Design

The rigid pavement design was carried out for the thickness of Jointed Plain Concrete Pavement (JPCP). In the calculations, the concrete class was taken as C35/45, and the plant-mix base thickness was assumed to be 20 cm, in accordance with the recommendations of the KGM Concrete Pavement Design Guide [54]. Furthermore, dowel and tie bars (reinforcements) were taken into account based on the same guide. At the end of 3, 7, and 28-day curing periods, the modulus of subgrade reaction (k) values were determined as follows: 378, 388, and 427 for untreated BT soil; and 604, 759, and 924 for 15% GGBFS-stabilized soil, respectively. Using the common values in Table 4 and Equation (3), the total layer thicknesses were calculated by considering the k values obtained at the end of the curing periods. All design parameters in Table 3, including the load transfer coefficient (J = 2.7 with doweled joints), drainage coefficient (C_d_ = 1.0), concrete modulus of rupture (S′c = 660 psi/4.5 MPa), and concrete modulus of elasticity (Ec = 4,786,244 psi/30,000 MPa), were selected based on KGM (2019) [43] guidelines to represent equivalent heavy traffic loading (200,000,000 ESALs) under regional climate and soil conditions, allowing a direct and rigorous methodological comparison between flexible and rigid pavement alternatives.

For the cost analysis of the rigid pavement, the unit weight (ton) costs for the base layer and unit volume (m^3^) costs for the concrete were based on the unit price schedules published by the KGM [81] and the Ministry of Environment, Urbanization, and Climate Change [82]. Depending on the layer thicknesses and the required reinforcement quantities determined in the design, the item numbers (pos numbers) used in the cost analysis are provided in Table 5.

**Figure 15:**
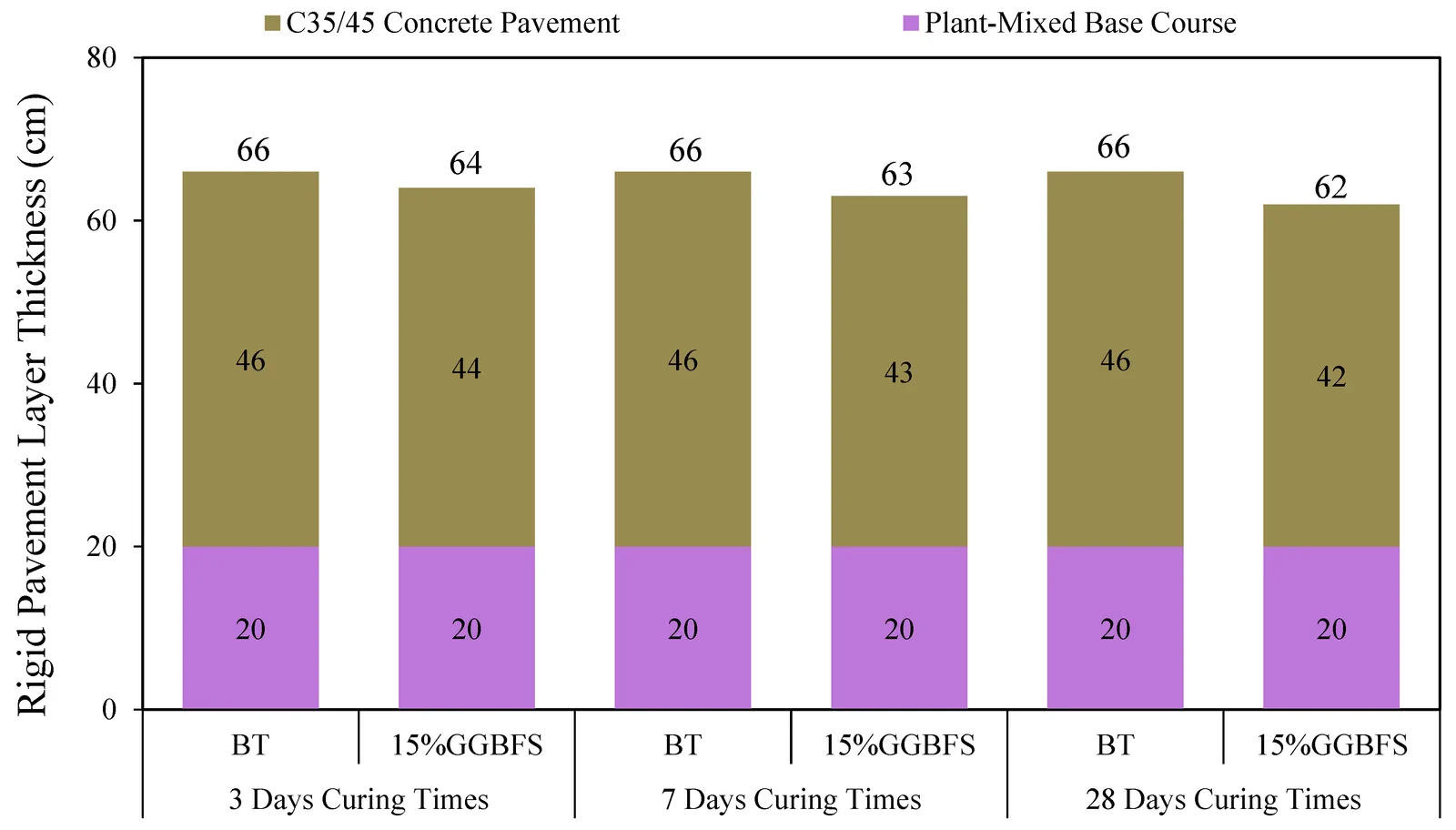
Layer thicknesses of rigid pavements of BT and GGBFS stabilized soils.

**Figure 16:**
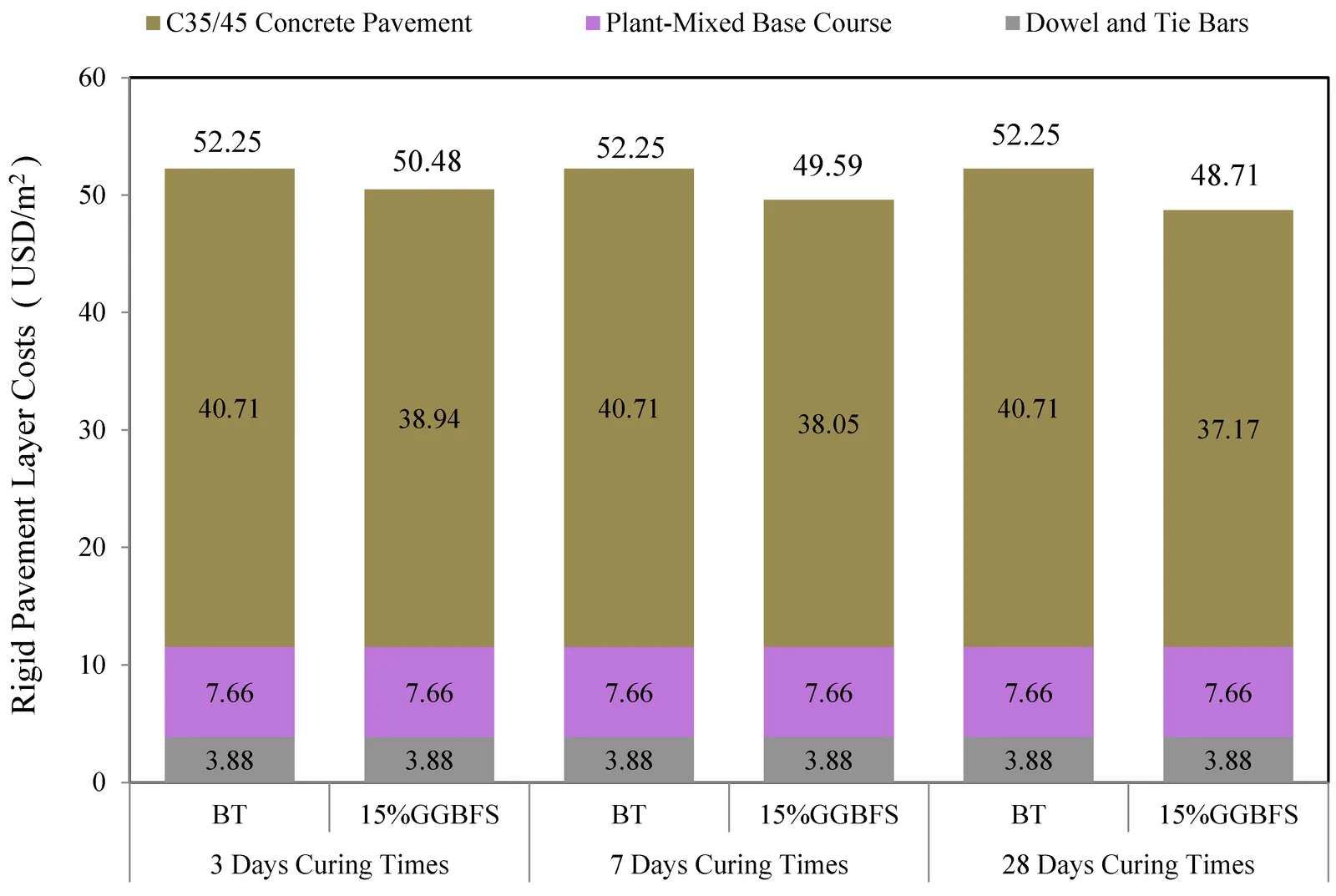
Layer costs of rigid pavements of BT and GGBFS stabilized soils.

In Figure 15 and Figure 16, the required concrete slab thicknesses for the untreated BT soil were calculated as 18.02 inches (45.77 cm), 17.97 inches (45.64 cm), and 17.92 inches (45.52 cm) for 3, 7, and 28-day curing periods, respectively; a thickness of 46 cm was selected for the analysis based on practical application. For the GGBFS-stabilized soil, the required concrete slab thicknesses were calculated as 17.31 inches (43.97 cm), 16.90 inches (42.93 cm), and 16.51 inches (41.94 cm), and thicknesses of 44, 43, and 42 cm were selected for the analysis based on practical application for the respective curing periods. Figures demonstrate that, compared to the untreated BT soil, the layer thicknesses of the GGBFS-stabilized soil decreased by 3.03%, 4.55%, and 6.06% for the different curing periods, respectively. The construction costs of rigid pavement using 15% GGBFS-stabilized soil decreased by 3.39%, 5.08%, and 6.77% per unit area (m^2^) compared to the untreated BT soil, depending on the curing periods. For a divided highway with dimensions of 1000 m × 20 m, this corresponds to savings of 35,396 USD, 53,094 USD, and 70,792 USD for the 3, 7, and 28-day curing periods, respectively.

### 3.8. Sustainability Analysis Based on CO_2_ Emissions During Initial Construction

**Figure 17:**
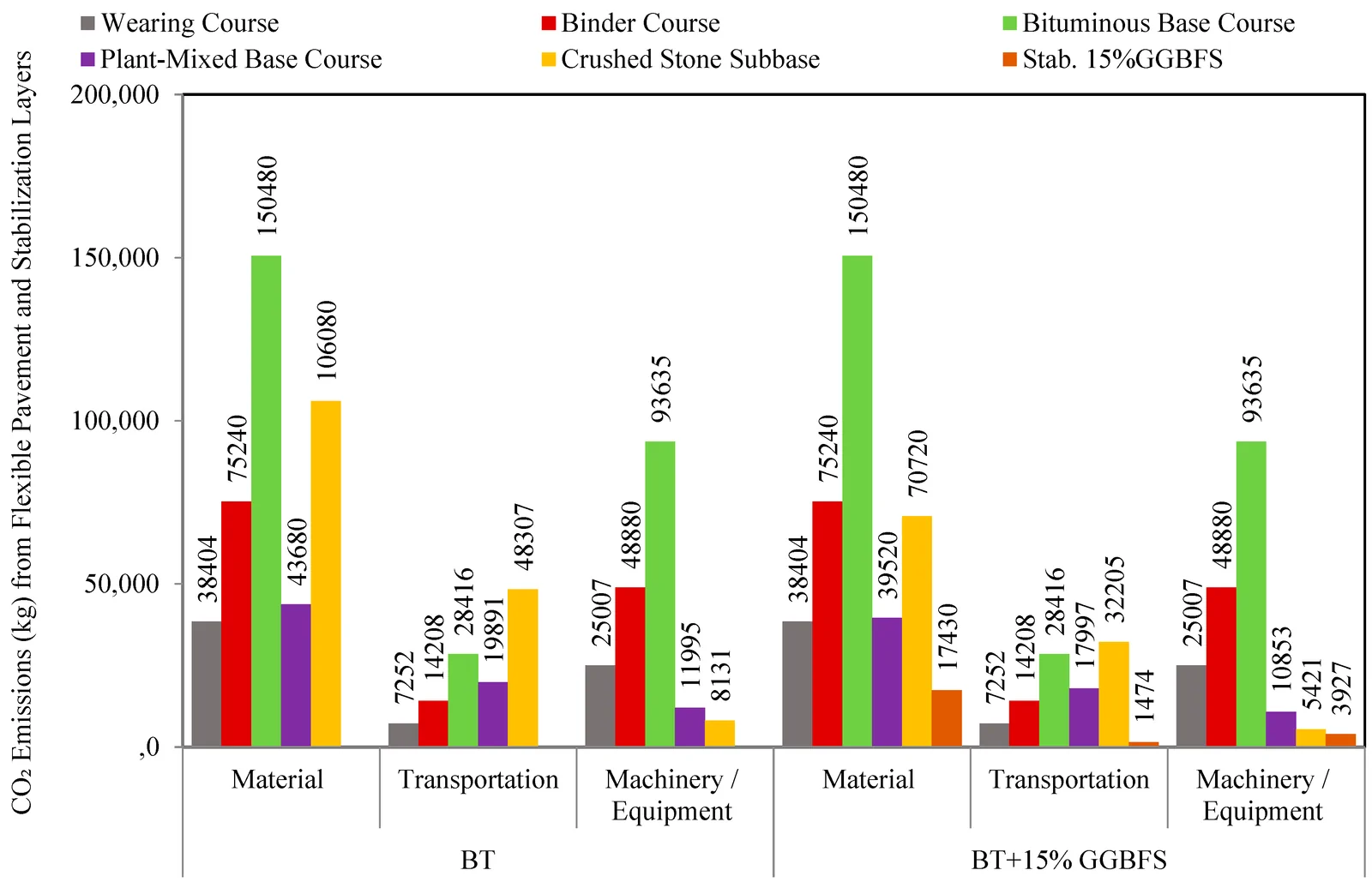
CO_2_ emissions of flexible pavement designs with BT and 15% GGBFS stabilized subgrade for 3 days curing period.

**Figure 18:**
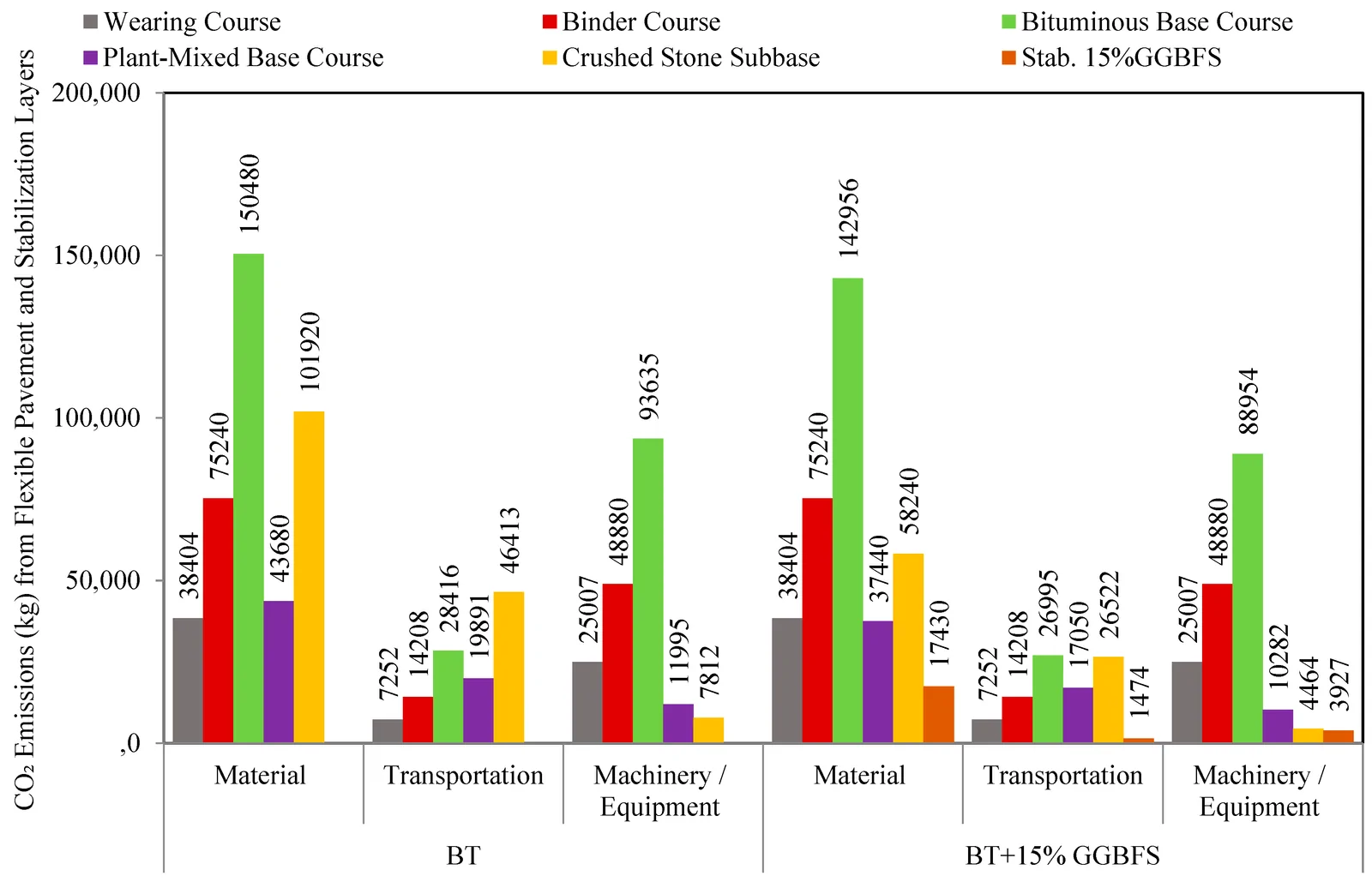
CO_2_ emissions of flexible pavement designs with BT and 15% GGBFS stabilized subgrade for 7 days curing period.

**Figure 19:**
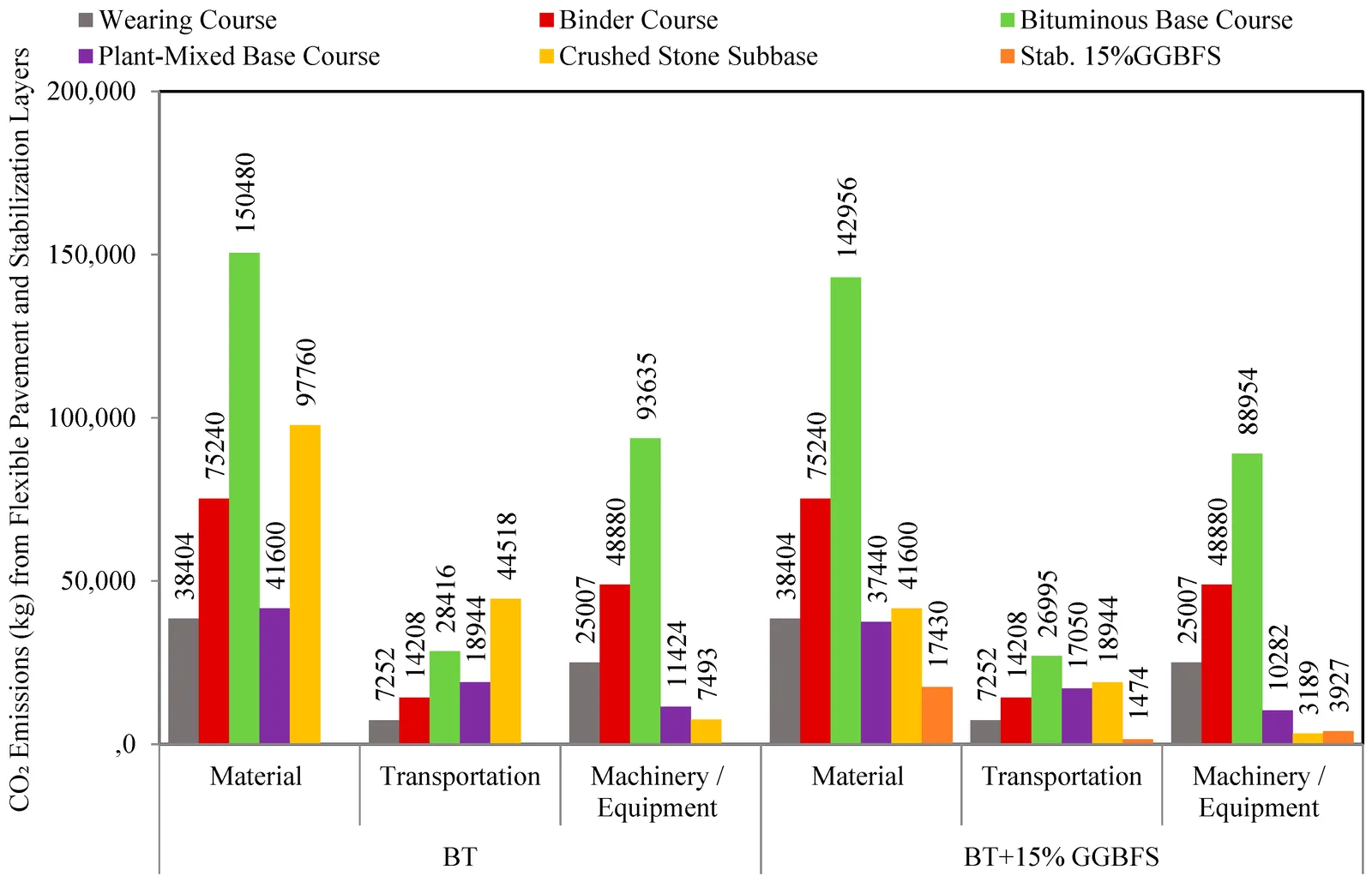
CO_2_ emissions of flexible pavement designs with BT and 15% GGBFS stabilized subgrade for 28 days curing period.

**Figure 20:**
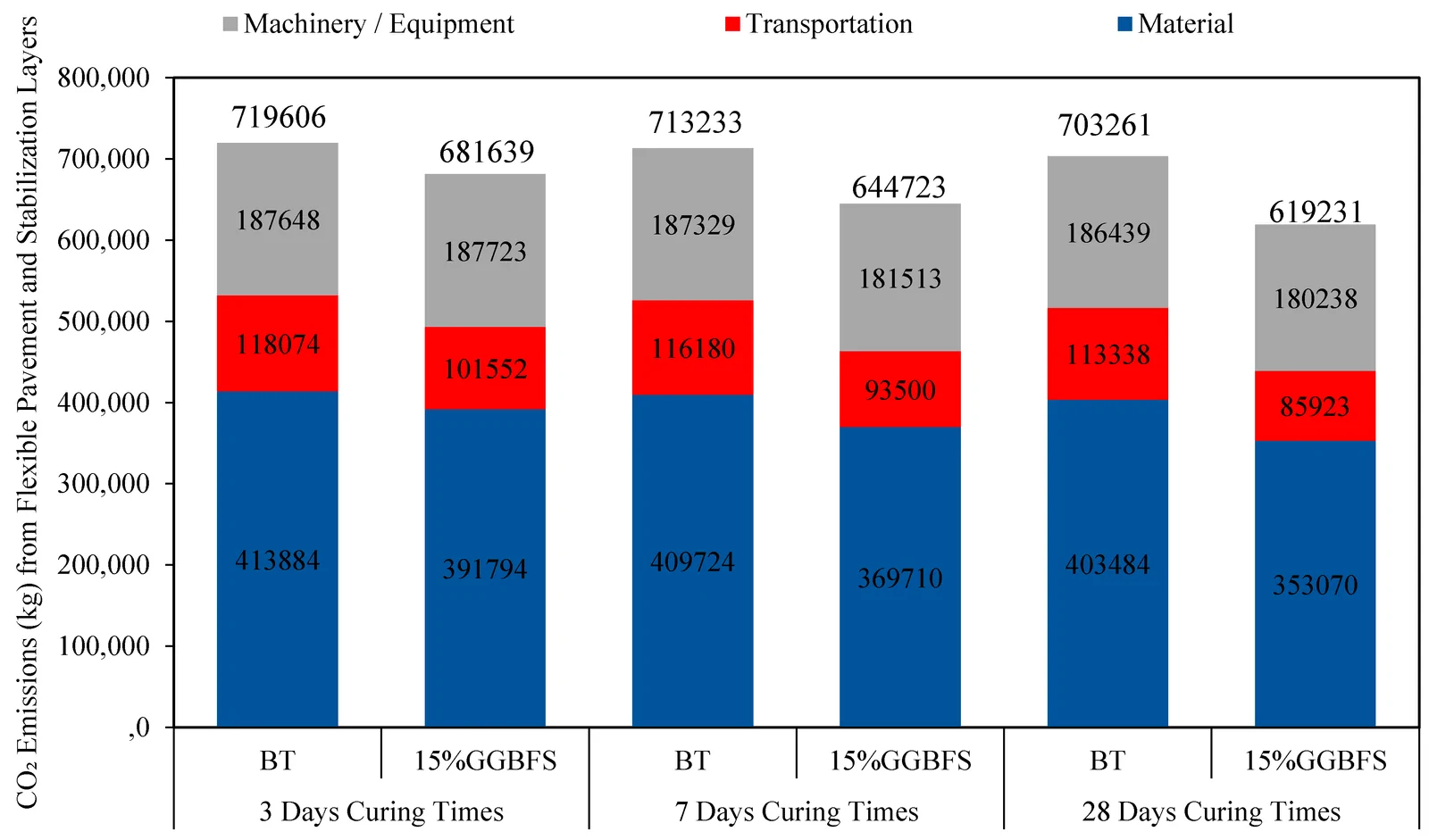
Comparison of CO_2_ emissions for flexible pavement designs with BT and 15% GGBFS stabilized subgrade.

Flexible and rigid pavement designs were developed based on the optimum mixture ratios obtained from tests conducted with untreated BT and GGBFS mixtures, considering 3, 7, and 28-day curing periods and a design traffic of 200,000,000 ESALs (Equivalent Single Axle Loads). In this context, CO_2_ emissions originating from materials, transportation, machinery, and equipment during the initial construction phase were compared between roads with untreated BT subgrades and roads stabilized using GGBFS. The prepared comparison graphs are based on the flexible and rigid pavement layers and the stabilization layer. Calculations were performed assuming 5% bitumen and 95% aggregate in bituminous layers. Furthermore, since dowel and tie reinforcements in rigid pavements are embedded within the concrete slab layer, the CO_2_ emissions of the concrete layer were included in the total value in the graphical representation. Using Equations (4) and (6), the study evaluated the CO_2_ emissions for flexible pavement designs containing untreated BT soil and soil stabilized with 15% GGBFS. Accordingly, the comparison of CO_2_ emissions generated during the initial construction phase of flexible pavements with untreated and 15% GGBFS-added subgrades is shown in Figure 17, Figure 18, Figure 19 and Figure 20.

Based on the investigation in Figure 17, Figure 18, Figure 19 and Figure 20 for a divided highway with dimensions of 1000 m × 20 m, the CO_2_ emissions originating from the initial construction phase were determined. In the design following a 3-day curing period, the flexible pavement with an untreated BT subgrade resulted in 413,884 kg from materials, 118,074 kg from transportation, and 187,648 kg from machinery and equipment, totaling 719,606 kg. The distribution of these emissions was 57.51% for materials, 16.41% for transportation, and 26.08% for machinery and equipment. For the flexible pavement design with a BT + 15% GGBFS subgrade, emissions were determined as 391,794 kg from materials, 101,552 kg from transportation, and 187,723 kg from machinery and equipment, totaling 681,639 kg. This distribution was 57.53% for materials, 14.91% for transportation, and 27.56% for machinery and equipment. The stabilization process achieved a 5.36% reduction in CO_2_ emissions. In the design after a 7-day curing period, the flexible pavement with an untreated BT subgrade totaled 713,233 kg (409,724 kg materials, 116,180 kg transportation, 187,329 kg machinery), with a distribution of 57.45%, 16.29%, and 26.26%, respectively. The design with a BT + 15% GGBFS subgrade totaled 644,723 kg (369,710 kg materials, 93,500 kg transportation, 181,513 kg machinery), with a distribution of 57.34%, 14.50%, and 28.15%. This stabilization resulted in a 9.61% reduction in CO_2_ emissions. In the design after a 28-day curing period, the flexible pavement with an untreated BT subgrade totaled 703,261 kg (403,484 kg materials, 113,338 kg transportation, 186,439 kg machinery), with a distribution of 57.37%, 16.12%, and 26.51%. The design with a BT + 15% GGBFS subgrade totaled 619,231 kg (353,070 kg materials, 85,923 kg transportation, 180,238 kg machinery), with a distribution of 57.02%, 13.88%, and 29.11%. This stabilization resulted in a 11.95% reduction in CO_2_ emissions.

**Figure 21:**
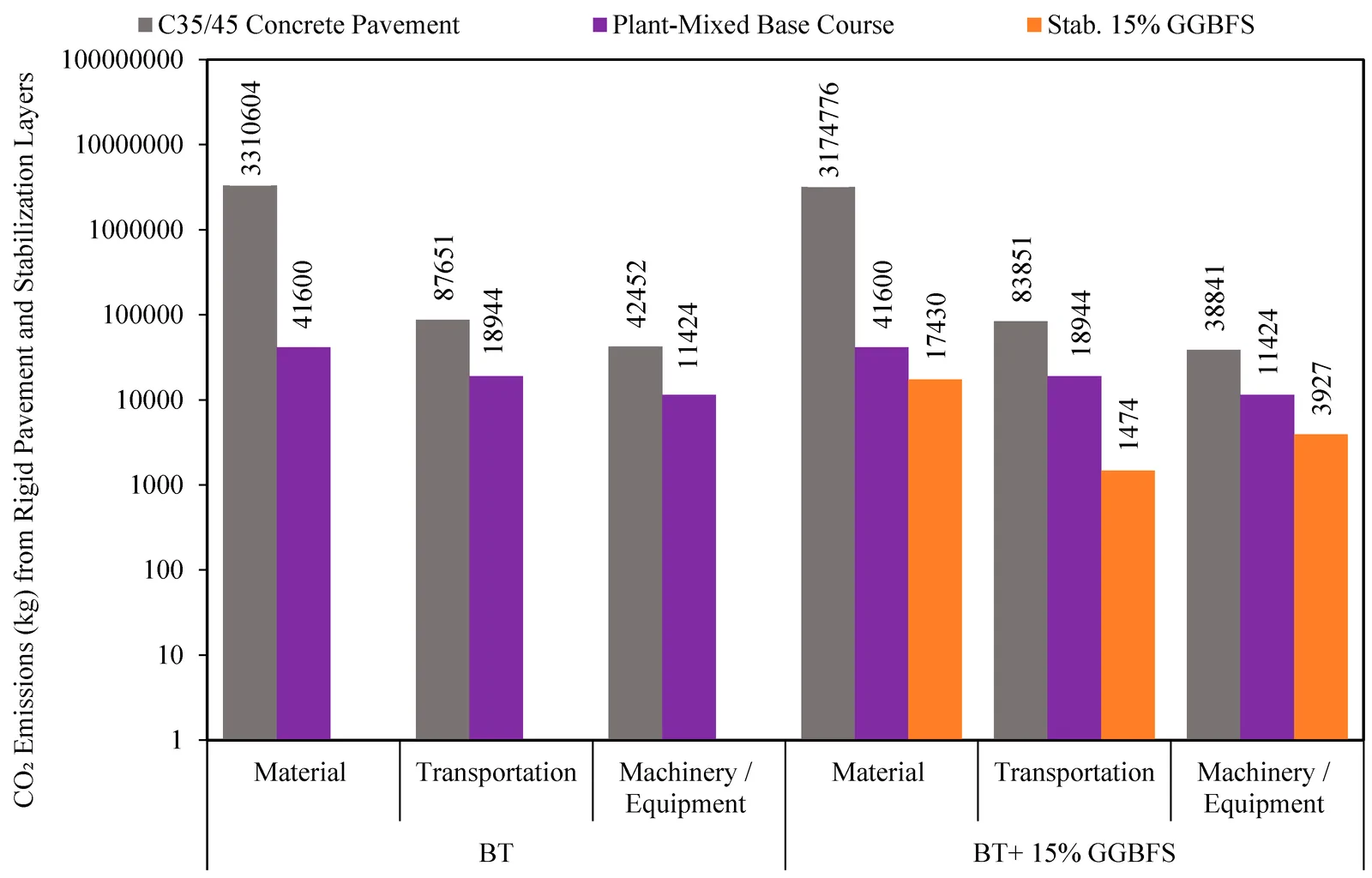
CO_2_ emissions of rigid pavement designs with BT and 15% GGBFS stabilized subgrade for 3 days curing period.

**Figure 22:**
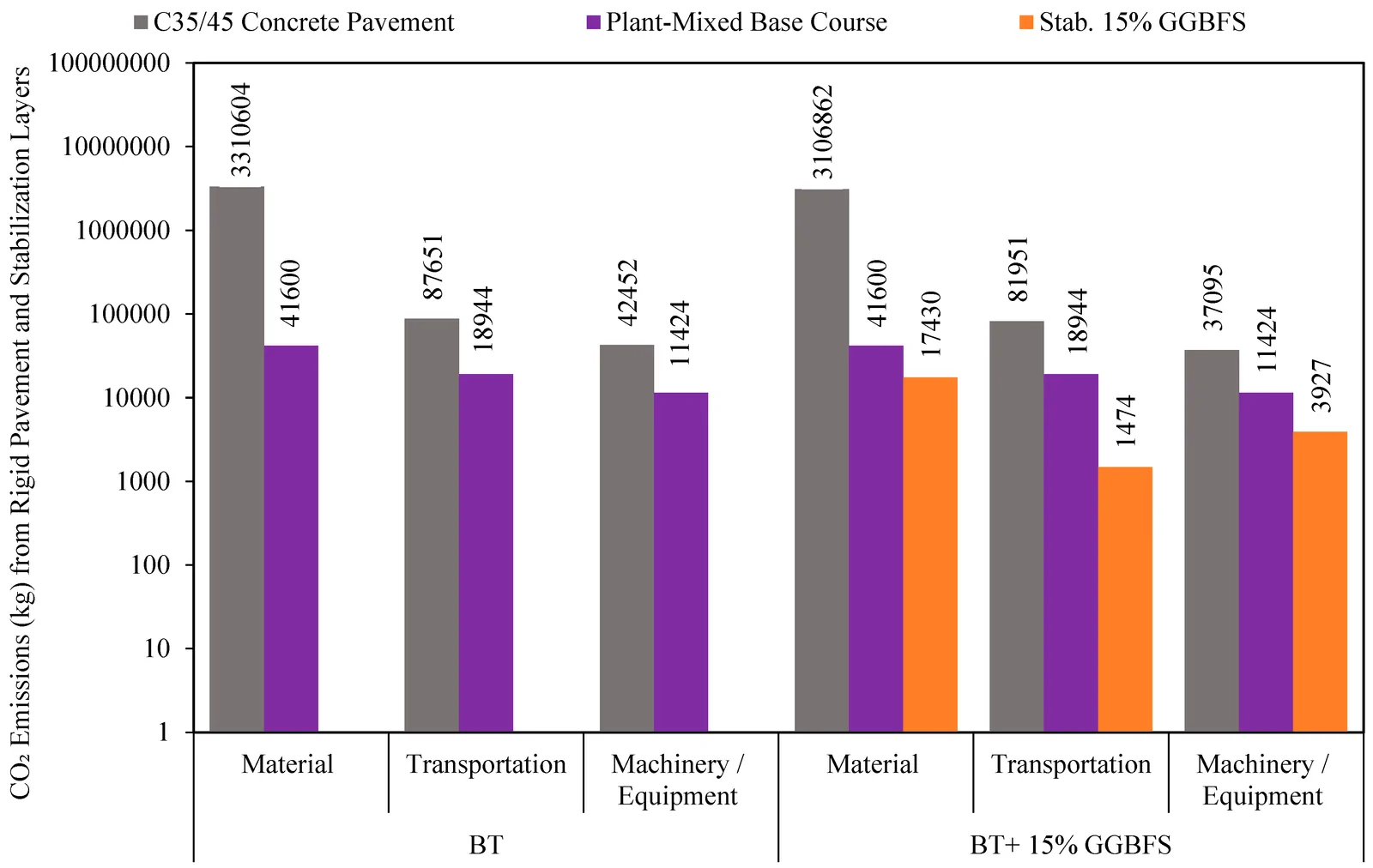
CO_2_ emissions of rigid pavement designs with BT and 15% GGBFS stabilized subgrade for 7 days curing period.

**Figure 23:**
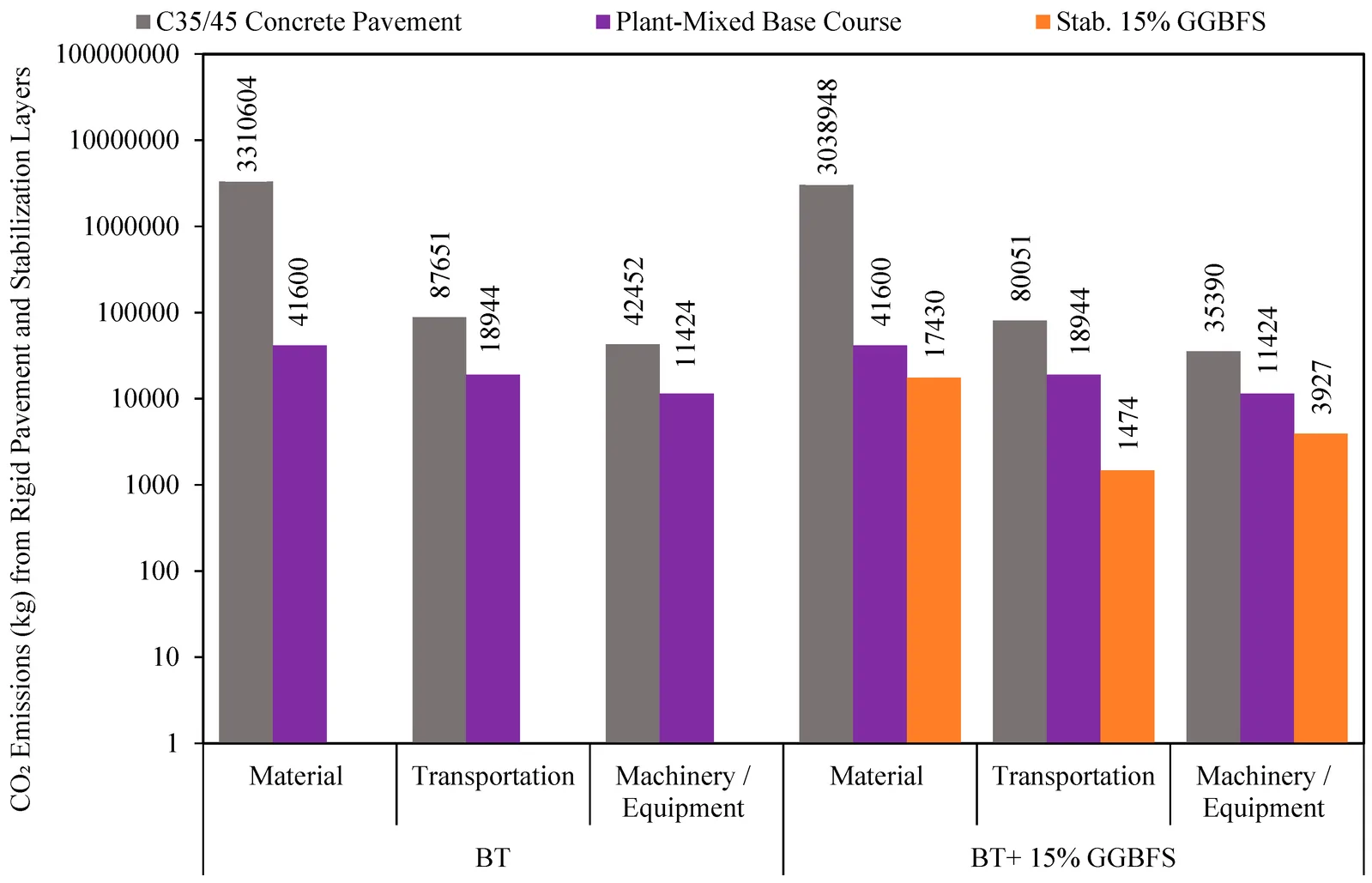
CO_2_ emissions of rigid pavement designs with BT and 15% GGBFS stabilized subgrade for 28 days curing period.

**Figure 24:**
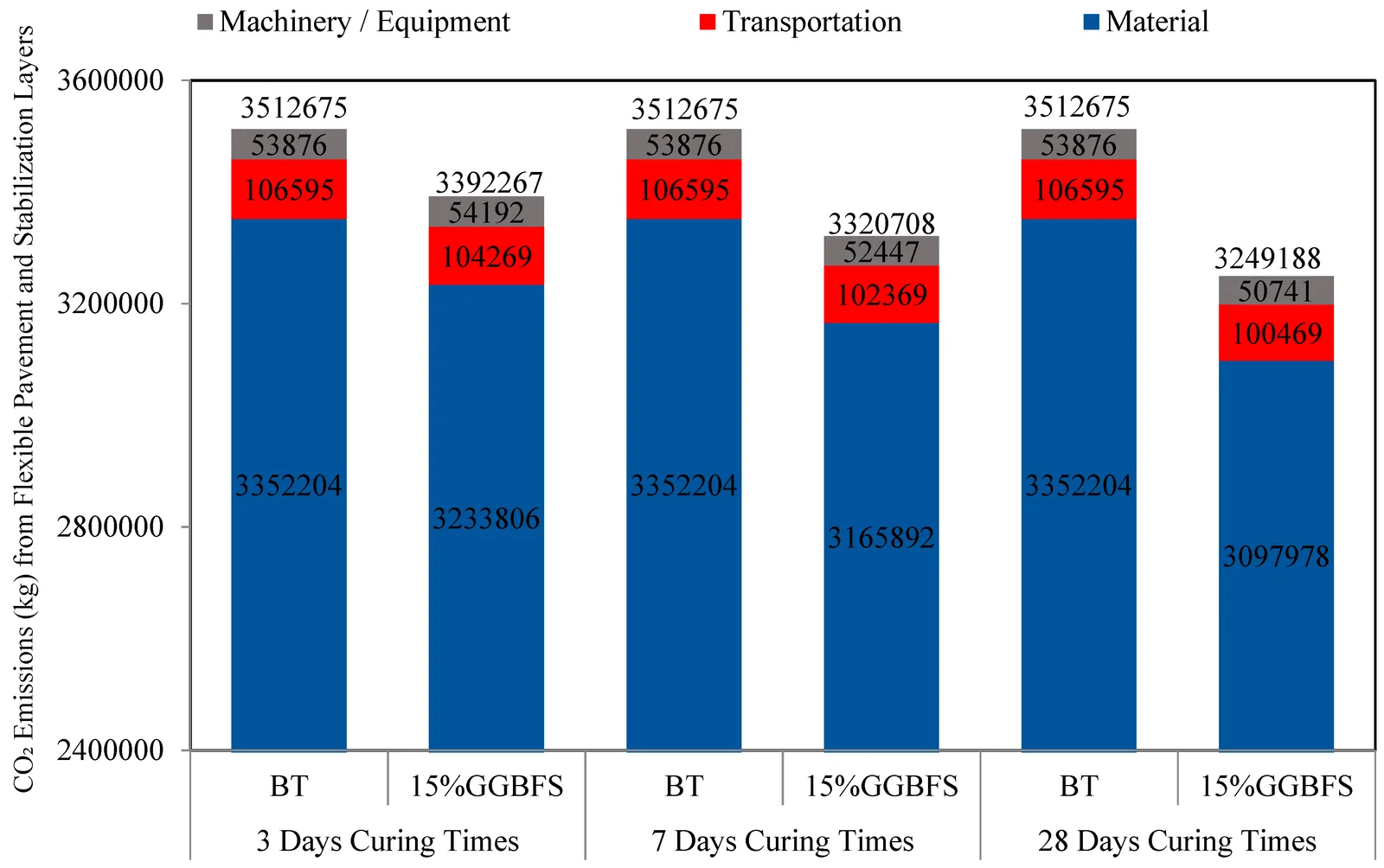
Comparison of CO_2_ emissions for rigid pavement designs with BT and 15% GGBFS stabilized subgrade.

Accordingly, the comparison of CO_2_ emissions generated during the initial construction phase of rigid pavements with untreated and 15% GGBFS-added subgrades is shown in Figure 21, Figure 22, Figure 23 and Figure 24.

Based on the investigation in Figure 20, Figure 21, Figure 22 and Figure 23 for a divided highway with dimensions of 1000 m × 20 m, the CO_2_ emissions originating from the initial construction phase were determined. Since the layer thicknesses remained the same for the designs at the end of 3, 7, and 28 day curing periods for the untreated BT subgrade, the rigid pavement design with an untreated BT subgrade resulted in 3,352,204 kg from materials, 106,595 kg from transportation, and 53,876 kg from machinery and equipment, totaling 3,512,675 kg. The distribution of these emissions was calculated as 95.43% for materials, 3.04% for transportation, and 1.53% for machinery and equipment.

In the design following a 3 day curing period, the rigid pavement with a BT + 15% GGBFS subgrade resulted in 3,233,806 kg from materials, 104,269 kg from transportation, and 54,192 kg from machinery and equipment, totaling 3,392,267 kg. The distribution of these emissions was 95.33% for materials, 3.07% for transportation, and 1.60% for machinery and equipment. The stabilization process achieved a 3.43% reduction in CO_2_ emissions. For the design after a 7-day curing period, the rigid pavement with a BT + 15% GGBFS subgrade resulted in 3,165,892 kg from materials, 102,369 kg from transportation, and 52,447 kg from machinery and equipment, totaling 3,320,708 kg. The distribution was 95.34% for materials, 3.08% for transportation, and 1.58% for machinery and equipment. This stabilization achieved a 5.46% reduction in CO_2_ emissions. In the design after a 28-day curing period, the rigid pavement with a BT + 15% GGBFS subgrade resulted in 3,097,978 kg from materials, 100,469 kg from transportation, and 50,741 kg from machinery and equipment, totaling 3,249,189 kg. The distribution was 95.35% for materials, 3.09% for transportation, and 1.56% for machinery and equipment. The stabilization process resulted in an 7.50% reduction in CO_2_ emissions. As a result of the calculations, it was observed that the primary source of total emissions is material-related (around 57% for flexible pavements and around 95% for rigid pavements), a finding that is consistent with the literature [36,37]. In light of these findings, reducing pavement layer thicknesses through stabilization is evaluated as an effective and appropriate approach.

The results obtained in this study indicate that soil stabilization significantly reduces initial construction costs and carbon dioxide emissions in both flexible and rigid pavement systems. Considering the substantial improvements in bearing capacity, resilient modulus, and freeze–thaw durability achieved through 15% GGBFS stabilization, it is expected that maintenance and rehabilitation requirements will decrease throughout the operational service life of the pavement. This finding aligns closely with established highway engineering principles reporting that pavements constructed on stabilized subgrades exhibit longer service life and lower structural maintenance demands [52,83]. Furthermore, according to the life cycle assessment principles defined in ISO 14040 and ISO 14044, reductions in maintenance activities directly contribute to lower cumulative CO_2_ emissions over time [84,85]. In this context, stabilization practices are projected to reduce material consumption, equipment operating hours, and traffic disruption during maintenance cycles, thereby further enhancing carbon emission reductions over the full pavement life cycle [52,85]. Consequently, to maintain a strict focus on the immediate feasibility and quantifiable structural impacts during the initial construction phase, additional detailed life cycle-based calculations of long-term maintenance and rehabilitation costs and operational carbon emissions were intentionally defined as outside the primary quantitative scope of this study [83,84,85].

## 4. Discussion

The observed improvements in the engineering performance of GGBFS-stabilized BT soil can be attributed to the progressive hydration and latent hydraulic reactions occurring during the curing period. These reactions promote the formation of cementitious products, such as calcium silicate hydrate (C-S-H) and calcium aluminosilicate hydrate (C-A-S-H), which strengthen interparticle bonding and reduce pore connectivity within the soil matrix [15]. Consequently, the stabilized soil exhibits greater stiffness and load-bearing capacity, which is consistent with the denser microstructure identified through SEM observations. This behavior indicates that the effectiveness of GGBFS stabilization depends not only on the replacement ratio but also on providing sufficient curing time for the stabilization reactions to develop.

From a pavement engineering perspective, the improved subgrade characteristics have implications that extend beyond the increase in laboratory strength parameters. Higher subgrade support enables the pavement structure to distribute traffic loads more efficiently, thereby reducing the structural demand on the overlying pavement layers [41]. This explains why improvements in stabilized soil properties were translated into reductions in pavement thickness rather than remaining solely as laboratory-scale performance gains. The more pronounced response observed in flexible pavements suggests that their structural design is more directly influenced by subgrade improvement than rigid pavement systems, where the concrete slab itself provides a greater proportion of the structural capacity.

The engineering improvements also produced indirect economic and environmental advantages through reductions in material demand. The primary economic benefit does not originate from the stabilization material itself but from the optimization of pavement geometry, which reduces the required quantities of aggregates, asphalt mixtures, and concrete during construction. Accordingly, reductions in material production, transportation, and placement contribute simultaneously to lower construction costs and lower embodied carbon emissions [30]. These findings emphasize the interconnected relationship between geotechnical improvement, pavement design optimization, and life-cycle sustainability.

Overall, the results demonstrate that the benefits of GGBFS stabilization should be evaluated using an integrated engineering framework rather than considering soil strength improvement as an isolated performance indicator. The interaction between subgrade enhancement, structural pavement response, material efficiency, and environmental performance highlights the importance of incorporating stabilized soil behavior into highway infrastructure design practices.

## 5. Conclusions

In this study, which investigated the effects of GGBFS-stabilized BT soil on highway pavements, the findings obtained from laboratory tests, pavement designs (flexible and rigid), cost analyses, and initial construction phase CO_2_ emission calculations are presented below:

1. Compaction Characteristics: According to Standard Proctor test results, increasing GGBFS replacement in mixtures prepared with BT soil raised the optimum moisture content and lowered the maximum dry unit weight. This is attributed to the increased water demand caused by the low specific gravity and porous texture of GGBFS.
2. Compressive Strength: Unconfined compressive strength results showed that increasing GGBFS content and curing periods enhanced the soil’s strength. Compared to untreated bentonite soil, 15% GGBFS-stabilized samples exhibited strength increases of 124%, 166%, and 208% at the end of 3, 7, and 28 days, respectively. SEM analyses confirmed that the improvement achieved with water and GGBFS resulted in a more compact structure compared to untreated soils.
3. Bearing Capacity: CBR test results for 15% GGBFS samples showed maximum increases of 109%, 185%, and 234% after 3, 7, and 28 days of curing, respectively. This indicates that the reactive nature of calcium, silica, and alumina in GGBFS enhances mechanical resistance by forming hydration products.
4. Curing Period Effect: The curing period was observed to be one of the most critical factors contributing to strength gain. In all cases, strength values increased as the curing period lengthened; therefore, determining an appropriate curing period is essential when designing pavement structures.
5. Freeze–Thaw Durability: Experimental durability assessments under 0, 3, 6, and 12 freeze–thaw cycles demonstrated that 15% GGBFS stabilization remarkably enhanced frost resistance. While untreated bentonite soil lost 73.74% of its compressive strength after 12 cycles (dropping to 22.11 kPa), the 15% GGBFS-stabilized soil retained a substantial residual strength of 83.53 kPa (a 278% performance advantage over untreated soil) with minimal mass loss (2.03%), confirming the effective formation of insoluble C-S-H gels against cyclic thermal stress.
6. Flexible Pavement Analysis: GGBFS stabilization reduced flexible pavement thickness by up to 29.41% and unit costs by up to 14.20%, resulting in considerable economic savings.
7. Rigid Pavement Analysis: For subgrades stabilized with 15% GGBFS, the total pavement thickness was reduced by up to 6.06%, while the unit area cost decreased by up to 6.77% compared with untreated BT soil. For a 1000 m long and 20 m wide divided highway, the implementation of GGBFS stabilization resulted in considerable economic savings, reaching up to 70,792 USD.
8. Environmental Impact: Comparison of initial construction CO_2_ emissions revealed that GGBFS stabilization reduced emissions by 5.36%, 9.61%, and 11.95% (representing 38,538 kg, 68,509 kg, and 84,031 kg of CO_2_ emission reduction) for flexible pavements. For rigid pavements, the reductions were 3.43%, 5.46%, and 7.50% (representing 120,408 kg, 191,967 kg, and 263,487 kg of CO_2_ emission reduction), respectively.

The CO_2_ savings achieved through GGBFS stabilization are equivalent to the annual CO_2_ sequestration capacity of thousands of mature trees, highlighting the significant potential for environmental sustainability in highway construction.

The findings of this study demonstrate that soil stabilization significantly improves the structural performance of both flexible and rigid pavement systems, leading to reductions in initial construction costs and carbon dioxide emissions. The enhanced bearing capacity and durability achieved through stabilization are expected to decrease maintenance and rehabilitation requirements throughout the pavement service life. As a result, stabilization practices contribute not only to short-term economic benefits but also to long-term environmental sustainability by reducing material consumption and associated carbon emissions. Therefore, soil stabilization can be considered an effective strategy for improving the overall economic and environmental performance of pavement infrastructure.

In conclusion, the use of GGBFS for soil stabilization is expected to provide significant reductions in pavement layer thicknesses and construction costs for both flexible and rigid pavement designs. These cost-effective designs are also projected to reduce CO_2_ emissions during the initial construction phase. Furthermore, this study highlights that increasing soil strength alone may not be sufficient to minimize highway construction costs. Since GGBFS is an industrial by-product with continuous availability, its utilization is expected to further enhance cost efficiency. In addition, by reducing the amount of material required for pavement construction through stabilization, the CO_2_ emissions associated with material production, transportation, and application can be minimized, thereby contributing to environmental sustainability.

Despite the promising findings, it should be acknowledged as a study limitation that the pavement thickness and performance evaluations in this research rely on CBR-based empirical correlations recommended in the AASHTO Mechanistic-Empirical Pavement Design Guide (MEPDG-3, 2024) for preliminary design-level analyses. While these practical correlations effectively capture general structural trends and facilitate comparative feasibility assessments, chemically stabilized soils may exhibit complex, stress-dependent, and non-linear deformation behavior in field applications. Furthermore, while 12-cycle laboratory freeze–thaw tests confirmed short-to-medium term environmental durability, long-term field performance under combined environmental exposure (wetting–drying cycles and moisture fluctuations) remains an essential requirement. Additionally, site-specific logistical variations, such as localized transport distances and regional material availability, should be considered when extending these construction-stage LCA findings to different geographic locations. Furthermore, the environmental sustainability analysis in this research specifically focuses on the initial construction phase (cradle-to-gate); evaluating operational maintenance and end-of-life phases remains a subject for future comprehensive life-cycle assessments. Most importantly, this study goes beyond conventional soil stabilization by emphasizing the strategic upcycling of GGBFS as an industrial waste material to directly minimize initial construction costs and cradle-to-gate CO_2_ emissions across both flexible and rigid pavement structures. Overall, this study serves as a preliminary research framework aimed at highlighting the significant potential, cost efficiency, and carbon footprint reduction benefits of GGBFS in pavement infrastructure. For detailed site-specific implementations, future studies utilizing direct cyclic triaxial resilient modulus testing, accelerated pavement testing, and long-term field performance monitoring are recommended to further refine these empirical estimates.

## Figures and Tables

**Table 1:** Percentage losses in unconfined compressive strength (UCS) and specimen weight of untreated BT soil and 15% GGBFS-stabilized soil following 3, 6, and 12 freeze-thaw cycles.

Samples	Test Results					
	UCS Loss (%)			Sample Weight Loss (%)		
	3 Cycles	6 Cycles	12 Cycles	3 Cycles	6 Cycles	12 Cycles
BT	35.89%	50.10%	73.74%	2.78%	3.23%	3.86%
BT + 15% GGBFS	28.51%	41.82%	66.51%	1.39%	1.67%	2.03%

**Table 2:** Parameters used in flexible pavement calculation.

Parameters	Selected Value
Equivalent standard axle load repetition number, W _8,2_	200,000,000
Combined standard error of traffic forecast and performance forecast, S_0_	0.45
Initial service index of road, P_0_	4.2
Final service index of road, P_t_	2.5
Total loss of service capability, Δ_PSI_	1.7
Standard normal deviation, Z_R_	−3.090

**Table 3:** Unit costs of layer thicknesses for flexible pavement.

Item No	Description	Unit	Unit Price
KGM/6405/S	5 cm asphalt concrete wearing course	m^2^	3.71 USD
KGM/6320	Asphalt concrete binder course	ton	27.65 USD
KGM/6220	Bituminous base course	ton	27.18 USD
KGM/6100	Plant-mixed base course	ton	15.95 USD
KGM/6000	Crushed stone sub-base course	m^3^	14.71 USD

**Table 4:** Parameters used in rigid pavement calculation.

Parameters	Selected Value
Equivalent standard axle load repetition number, W _8,2_	200,000,000
Load transfer coefficient, J	2.7
Drainage coefficient, C_d_	1
Modulus of rupture of concrete (psi), S′c	660
Compound standard error of traffic forecast and performance forecast, S_0_	0.35
Initial service index, P_0_	4.5
Final service index of road, Pt (For motorways, state roads 2.5)	2.5
Total loss of service capability, Δ_PSI_	2
Modulus of elasticity of concrete, E_c_ (C 35/45) (psi)	4,786,244
Standard normal deviation, Z_R_	−3.090

**Table 5:** Unit costs of layer thicknesses for rigid pavement.

Item No	Description	Unit	Unit Price
15.150.1007	Concrete Slab (C35/45)	m^3^	88.49 USD
KGM/6100	Plant-mixed base course	ton	15.95 USD
15.160.1004	Reinforcing Steel Bars Ø 14–28 mm	ton	1030.47 USD
15.160.1005	Reinforcing Steel Bars Larger than Ø 28 mm	ton	1008.99 USD

Ø: diameter.

## Data Availability

The data presented in this study are available from the corresponding author upon reasonable request. The data are not publicly available because they form part of an ongoing research project.

## Abbreviations

The following abbreviations are used in this manuscript:

SEM	Scanning electron microscope
CH	High plasticity clay
GGBFS	Ground granulated blast furnace slag
BT	Bentonite
CBR	California bearing ratio
USD	United States dollar
ASTM	American society for testing and materials
MR	Effective modulus of subgrade reaction for the subgrade soil (psi)
SN	Structural number (used for pavement layer thickness calculation)
ΔPSI	Total serviceability loss
